# State-of-the-art analytical methods of viral infections in human lung organoids

**DOI:** 10.1371/journal.pone.0276115

**Published:** 2022-12-20

**Authors:** Morris Baumgardt, Maren Hülsemann, Anna Löwa, Diana Fatykhova, Karen Hoffmann, Mirjana Kessler, Maren Mieth, Katharina Hellwig, Doris Frey, Alina Langenhagen, Anne Voss, Benedikt Obermayer, Emanuel Wyler, Simon Dökel, Achim D. Gruber, Ulf Tölch, Stefan Hippenstiel, Andreas C. Hocke, Katja Hönzke

**Affiliations:** 1 Department of Infectious Diseases and Respiratory Medicine, Charité–Universitätsmedizin Berlin, Corporate Member of Freie Universität Berlin and Humboldt Universität zu Berlin, Berlin, Germany; 2 Berlin Institute of Health at Charité (BIH), BIH QUEST Center for Responsible Research, Berlin, Germany; 3 Department of Gynecology and Obstetrics, University Hospital, LMU, Munich, Germany; 4 Department of Veterinary Pathology, Freie Universität Berlin, Berlin, Germany; 5 Core Unit Bioinformatics, Berlin Institute of Health at Charité–Universitätsmedizin Berlin, Berlin, Germany; 6 Berlin Institute for Medical Systems Biology (BIMSB), Max Delbrück Center for Molecular Medicine in the Helmholtz Association (MDC) and IRI Life Sciences, Institute for Biology, Humboldt Universität zu Berlin, Berlin, Germany; University of Helsinki: Helsingin Yliopisto, FINLAND

## Abstract

Human-based organ models can provide strong predictive value to investigate the tropism, virulence, and replication kinetics of viral pathogens. Currently, such models have received widespread attention in the study of SARS-CoV-2 causing the COVID-19 pandemic. Applicable to a large set of organoid models and viruses, we provide a step-by-step work instruction for the infection of human alveolar-like organoids with SARS-CoV-2 in this protocol collection. We also prepared a detailed description on state-of-the-art methodologies to assess the infection impact and the analysis of relevant host factors in organoids. This protocol collection consists of five different sets of protocols. Set 1 describes the protein extraction from human alveolar-like organoids and the determination of protein expression of angiotensin-converting enzyme 2 (ACE2), transmembrane serine protease 2 (TMPRSS2) and FURIN as exemplary host factors of SARS-CoV-2. Set 2 provides detailed guidance on the extraction of RNA from human alveolar-like organoids and the subsequent qPCR to quantify the expression level of *ACE2*, *TMPRSS2*, and *FURIN* as host factors of SARS-CoV-2 on the mRNA level. Protocol set 3 contains an in-depth explanation on how to infect human alveolar-like organoids with SARS-CoV-2 and how to quantify the viral replication by plaque assay and viral E gene-based RT-qPCR. Set 4 provides a step-by-step protocol for the isolation of single cells from infected human alveolar-like organoids for further processing in single-cell RNA sequencing or flow cytometry. Set 5 presents a detailed protocol on how to perform the fixation of human alveolar-like organoids and guides through all steps of immunohistochemistry and *in situ* hybridization to visualize SARS-CoV-2 and its host factors. The infection and all subsequent analytical methods have been successfully validated by biological replications with human alveolar-like organoids based on material from different donors.

## Introduction

The current outbreak of the COVID-19 pandemic caused by the severe acute respiratory syndrome coronavirus type 2 (SARS-CoV-2) has once again highlighted the need for appropriate and rapidly available model systems with high predictive value towards clinical translation [1]. The SARS-CoV-2 exposure of most animal models either did not lead to infection, or only partly reflect relevant human aspects of disease [2–4]. Hence, human-based organ models provide promising opportunities for the rapid study of pathogens and their impact on the human system [5–10]. To investigate the effects of a viral pathogen on our respiratory system, human alveolar-like organoids serve as an excellent tool to gain insights on virulence mechanisms and viral tropisms [11]. However, standard infection methods used in classical 2D cell culture systems are not transferable to 3D human alveolar-like organoids [12].

Therefore, we are providing a detailed protocol collection on the infection of human alveolar-like organoids as well as their preparation for associated readouts. The protocol collection includes an in-depth instruction on the handling and preparation of human alveolar-like organoids for infection with SARS-CoV-2 under biosafety level 3 conditions, followed by the measurement of viral replication by plaque assay and viral quantitative reverse transcription PCR (RT-qPCR) [13].

This protocol collection also provides the thorough work instructions to determine the expression of host factors in 3D organ models on the protein and mRNA level. Exemplarily, the expression of ACE2, TMPRSS2, and FURIN as key host factors of SARS-CoV-2 is described by Western blotting and real-time quantitative PCR (qPCR) [14, 15]. Novel technologies such as single-cell RNA sequencing (scRNA-seq) have tremendous impact on current COVID-19 research. Applied on human alveolar-like organoids, scRNA-seq can increase our understanding on the pathophysiological signature, and the cell-mediated immune response after SARS-CoV-2 infection. Therefore, this protocol collection includes a detailed description of single cell preparation from human alveolar-like organoids for further processing in single-cell technologies. Imaging techniques such as immunohistochemistry and *in situ* hybridization allow a detailed analysis of viral distribution, tissue damage and host factor location in the human alveolar-like organoids. We provide elaborate instructions on sample preparation and staining procedures to provide sections for high resolution imaging [16].

Given the urgency of the pandemic, researchers worldwide need to coordinate their activities and support collaborative efforts in COVID-19 research. Thereby, the distribution of transparent and detailed protocols is an essential tool towards high reproducibility rates and scientific rigor [17–19].

## Materials and methods

The protocol collection described in this peer-reviewed article is published on protocols.io (doi.org/10.17504/protocols.io.5jyl89pw6v2w/v2) and is attached to this article as S1–S6 Files for printing. Informed written consent was obtained from all volunteers and the study was approved by the Charité Ethics Committee (project 451, EA2/079/13).

### Expected results

The presented protocols enabled us to screen mature human alveolar-like organoids [11] for three important currently known host factors of SARS-CoV-2.

**Fig 1:**
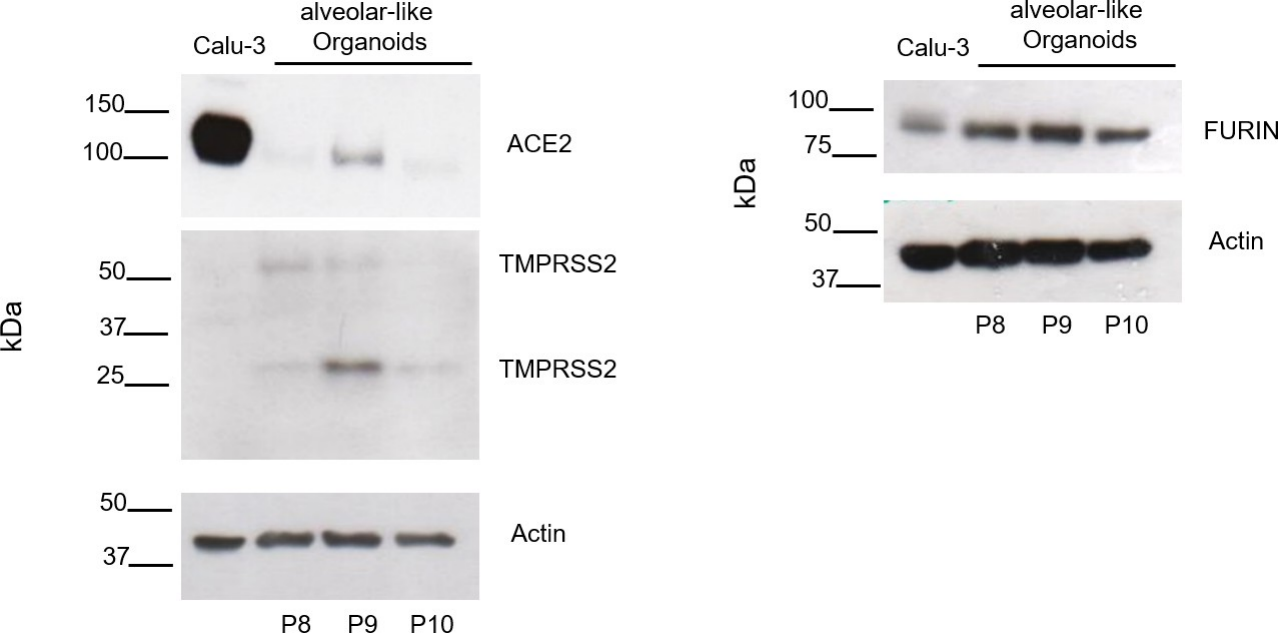
Analysis of SARS-CoV-2 host factor expression by Western blot. Representative Western blots for ACE2, TMPRSS2, and FURIN of protein lysates from human alveolar-like organoids of three different donors (P8-P10). Calu-3 cells served as positive control.

**Fig 2:**
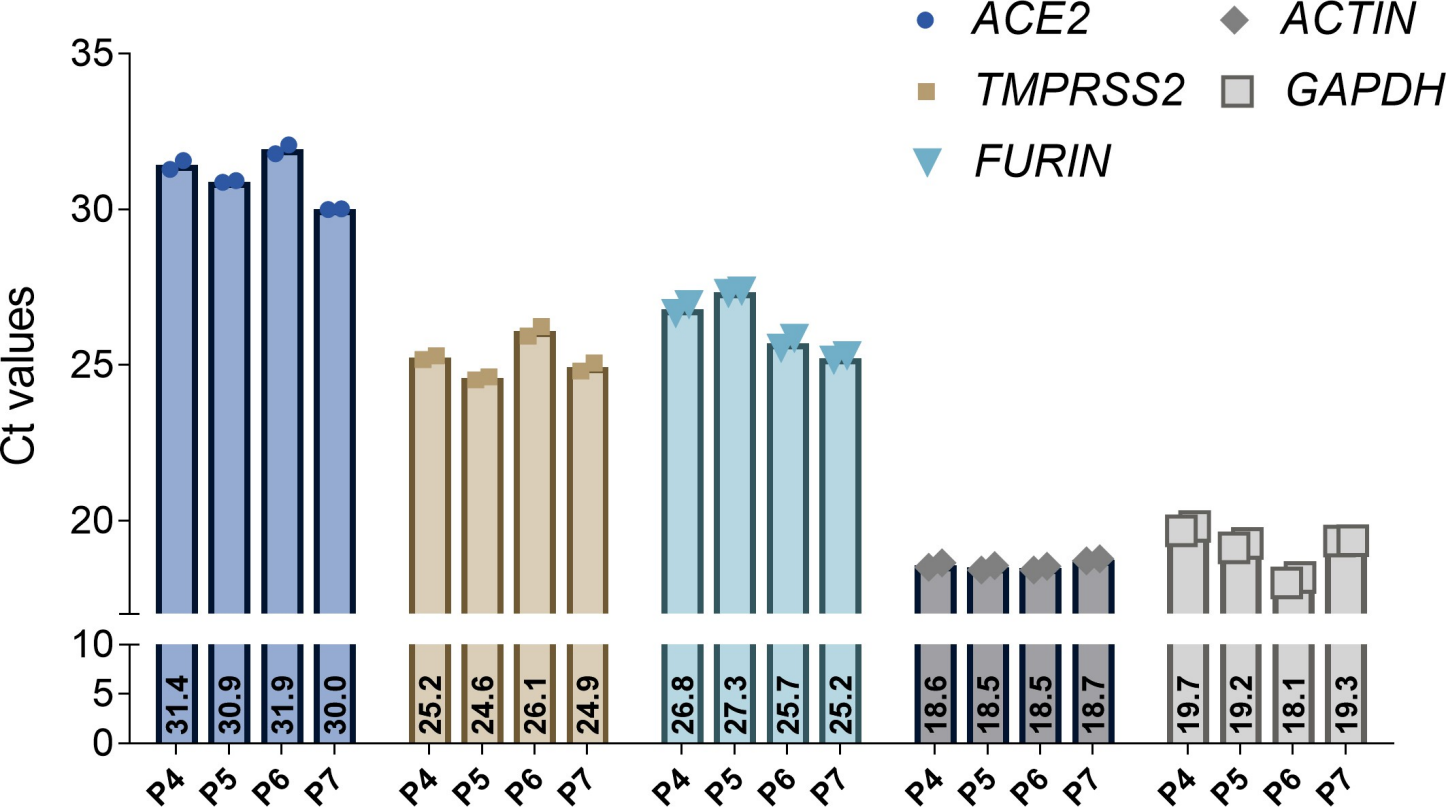
Analysis of *ACE2*, *TMPRSS2*, and *FURIN* expression in human alveolar-like organoids. Expression of *ACE2*, *TMPRSS2*, *FURIN* and *GAPDH* and β*-ACTIN* as housekeeping genes measured by qPCR on bulk RNA of human alveolar-like organoids. Shown are Ct values of four different donors (P4-P7) and two technical replicates.

We analyzed ACE2, TMPRSS2 and FURIN in human alveolar-like organoids (n = 3) as exemplary host factors by Western blot and used Calu-3 cells as positive control (Fig 1). All three host factors were expressed in the alveolar-like organoids. The TMPRSS2 blot showed two characteristic bands described as full-length TMPRSS2 (54kDa) and the cleaved form (25kDa). The mRNA expression level of SARS-CoV-2 host factors *ACE2*, *TMPRSS2*, *and FURIN* were analyzed by qPCR in human alveolar-like organoids (n = 4) (Fig 2). The figure shows the basic expression of the host factors by using Ct values and two different reference genes (*GAPDH*, β*-ACTIN*).

**Fig 3:**
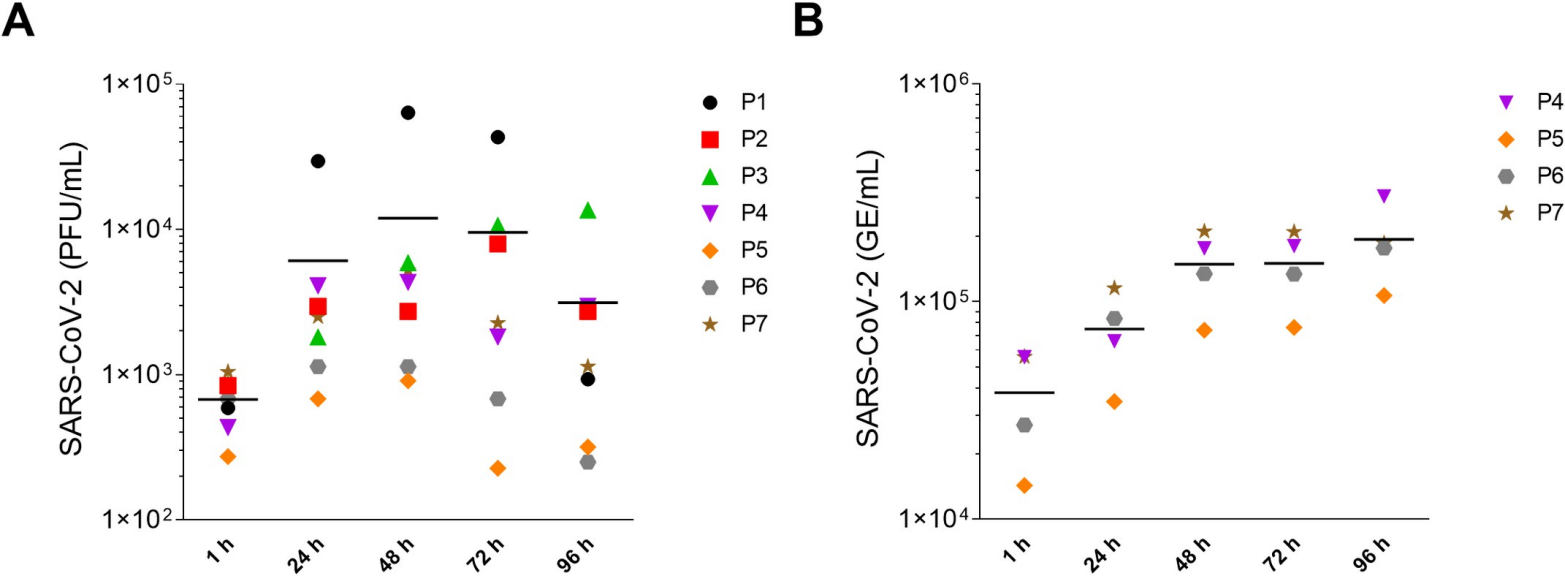
Replication kinetics of SARS-CoV-2-infected human alveolar-like organoids. Human alveolar-like organoids were infected with SARS-CoV-2 (MOI = 1) and viral replication was assessed by plaque assay (A) and via viral E gene-based RT-qPCR (B). Data of seven (plaque assay) and four (RT-qPCR) biological replicates are shown as individual data points. The mean is visualized by a horizontal black line.

The successful infection of human alveolar-like organoids with SARS-CoV-2 was shown by the increasing number of infectious particles over the time course of 96 hours for seven different donors via plaque assay (Fig 3A). Additionally, the number of viral genomic copies was quantified in the cell culture supernatant via viral E gene-based RT-qPCR, as described in the protocol collection (Fig 3B). The viral RNA that is produced indicates whether viral replication occurs and virions are formed. However, it is not possible to say whether these viral particles are complete and infectious. Therefore, it is of particular importance to also generate a replication curve via the plaque assay approach, which shows the number of infectious particles over time. Especially when it comes to drug screening, the indication of viral RNA is not sufficient. In that sense, the two values are not comparable, but together they give a good indication of the overall replication dynamics.

**Fig 4:**
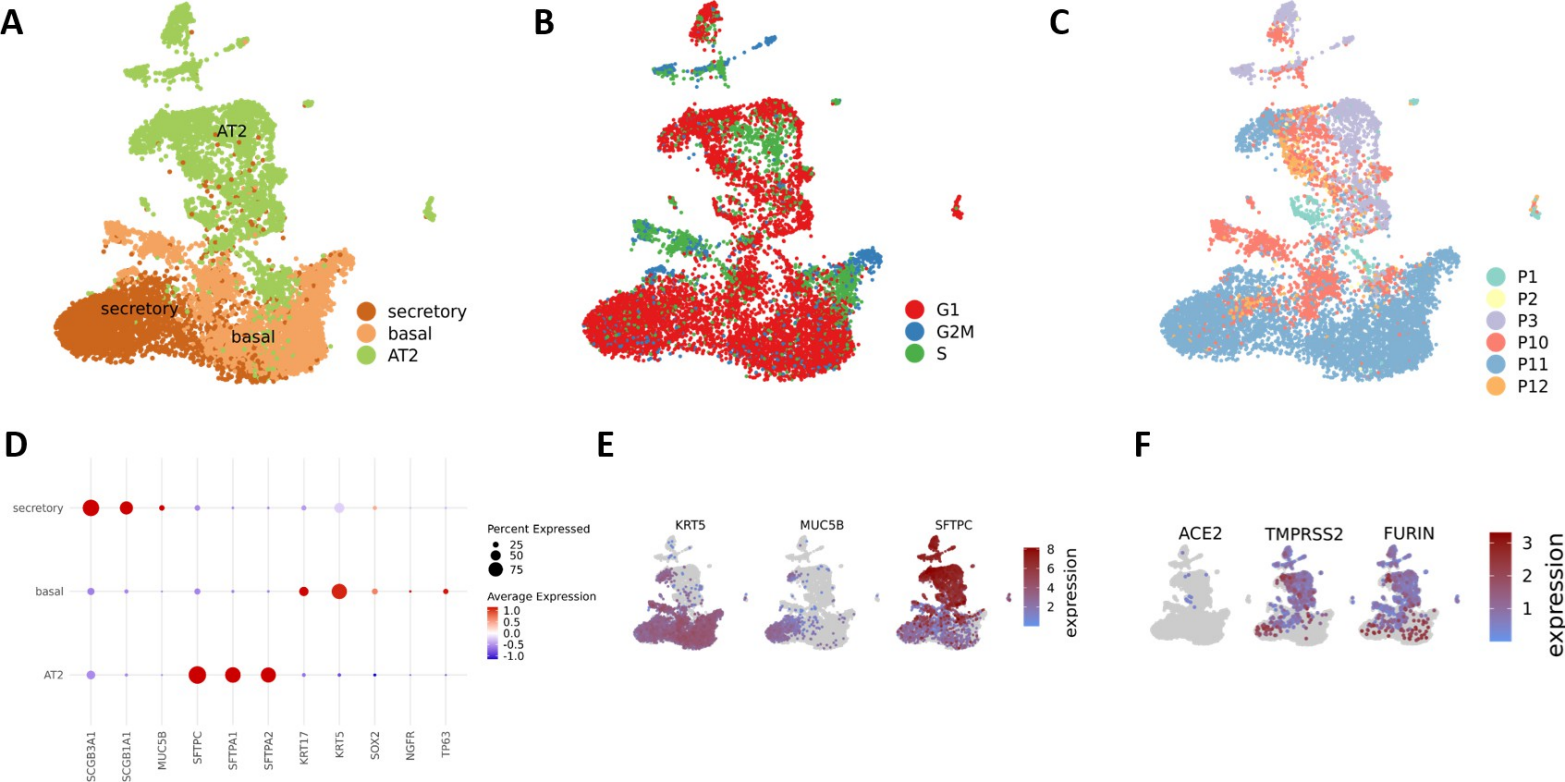
Single-cell RNA sequencing displays the proportion of three different cell types and SARS-CoV-2 host factors present in uninfected human alveolar-like organoids. UMAP embedding of human alveolar-like organoids (n = 6; P1-P3 and P10-P12) shows AT2, basal, and secretory cells present in the organoids (A). Cell cycle phase of each cell in either G2M, S or G1 phase (B) and individual donor composition (C) is displayed. The marker genes needed for cell type identification are shown (D and E) as well as the expression of the SARS-CoV-2 host factors *ACE2*, *TMPRSS2*, and *FURIN* (F).

After enzymatic digestion of human alveolar-like organoids into single cell suspension, scRNA-seq can be performed to allow dissection of gene expression at single-cell resolution (Fig 4). In this exemplary case, scRNA-seq data from human alveolar-like organoids of six donors were annotated according to Travaglini et al. [20] and the following cell clusters were identified: AT2, basal and secretory cells (Fig 4A). The markers used for cell type identification are shown in Fig 4D and 4E. The used protocol generates organoid cultures of the alveolar epithelium from bipotent progenitors excluding at least in part bronchial cells. AT1 cells were not found in the model, the mechanism underlying their differentiation is not completely understood and needs further elucidation. Fig 4B shows the specific cell cycle phases. Most of the cells are in the G1-phase which is believed to serve as a window of opportunity to initiate cell differentiation [21]. The UMAP in Fig 4C shows the different donors combined in this study. ScRNA-seq enables an in-depth analysis of the effect of SARS-CoV-2 infection on the different cell types and allows validation of their host factor expression levels as shown in Fig 4F for *ACE2*, *TMPRSS2*, and *FURIN*. These expression patterns are consistent with those already observed at the protein and mRNA levels (Figs 1 and 2). All data are available at: https://ncbi.nlm.nih.gov/geo/query/acc.cgi?acc=GSE197949 and https://ncbi.nlm.nih.gov/geo/query/acc.cgi?acc=GSE198864.

**Fig 5:**
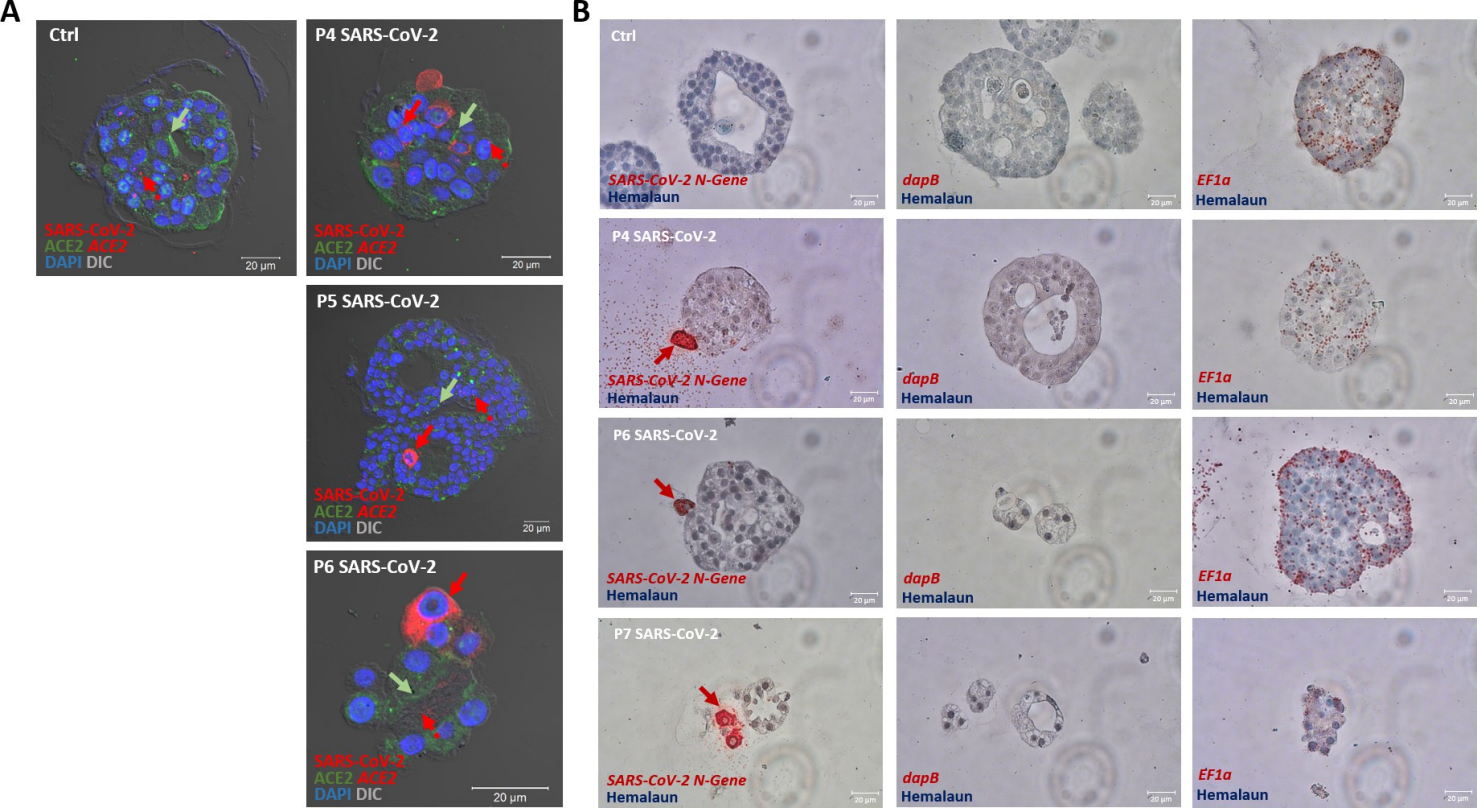
Spectral imaging of mock- and SARS-CoV-2-infected and immunostained human alveolar-like organoids and *in situ* hybridization. (A) Representative immunostainings for exemplary mock- (left panel) and SARS-CoV-2-infected (right column) human alveolar-like organoids. Shown are immunostainings for SARS-CoV-2 (N-protein, red), ACE2 (green) and via *in situ* hybridization visualized *ACE2* mRNA expression (red dots) 24 h post infection (MOI = 1). Arrows indicate either cells positive for SARS-CoV-2 (red arrows) or areas of particularly high ACE2 expression (protein: green arrows, mRNA: red dotted arrows). Cell nuclei are visualized by DAPI stain (blue). Scale bars = 20 μm. (B) *In situ* hybridization of human alveolar-like organoids shows SARS-CoV-2 mRNA expression (left column). *DapB* and *EF1a* served as negative respectively positive control. Red arrows indicate cells positive for SARS-CoV-2. Scale bars = 20 μm.

In order to visualize the expression patterns of host factors and the virus, we utilized immunohistochemistry (n = 3) and *in situ* hybridization (n = 3) and acquired high-resolution imaging data showing viral particles that have migrated into human alveolar-like organoids as well as ACE2 expression (Fig 5). *In situ* hybridization also shows viral particles present in alveolar-like organoids and *ACE2* expression (Fig 5). In context of this protocol collection, we present exemplary data, however, experiments should be performed on at least three biological replicates of human alveolar-like organoids derived from different donors.

## Supporting information

S1 File Collection: State-of-the-art analytical methods of viral infections in human lung organoids. (PDF)

S2 File Step-by-step protocol: Protein extraction and western blot of human lung organoids. (PDF)

S3 File Step-by-step protocol: RNA Extraction and RT-qPCR of human lung organoids. (PDF)

S4 File Step-by-step protocol: SARS-CoV-2 infection and viral replication of human lung organoids. (PDF)

S5 File Step-by-step protocol: Single cell isolation of human lung organoids. (PDF)

S6 File Step-by-step protocol: Fixation, immunohistochemistry and *in situ* hybridization of human lung organoids. (PDF)

S1 Raw images Uncropped and unadjusted blots. (TIF)
