# Correction: State-of-the-art analytical methods of viral infections in human lung organoids

**DOI:** 10.1371/journal.pone.0294216

**Published:** 2023-11-03

**Authors:** Morris Baumgardt, Maren Hülsemann, Anna Löwa, Diana Fatykhova, Karen Hoffmann, Mirjana Kessler, Maren Mieth, Katharina Hellwig, Doris Frey, Alina Langenhagen, Anne Voss, Benedikt Obermayer, Emanuel Wyler, Simon Dökel, Achim D. Gruber, Ulf Tölch, Stefan Hippenstiel, Andreas C. Hocke, Katja Hönzke

There are errors in the Funding section. The correct Funding statement is: K.H., A.L., M.K., K.H., A.C.H., S.H., M.H., U.T., A.D.G., S.D. and M.B. were supported by Einstein Foundation (EC3R EZ-2020-597-2, [http://www.einsteinfoundation.de)]http://www.einsteinfoundation.de) K.H., B.O., S.H., A.D.G., S.D. and A.C.H. were funded by BMBF (Organo-Strat 01KX2021, https://www.bmbf.de/bmbf/de/home/home_node.html) E.W. was funded by Ministry of Education and Research (https://www.bmbf.de/bmbf/de/home/home_node.html) A.C.H. and S.H. were supported by Berlin University Alliance GC2 Global Health (Corona Virus Pre-Exploration Project, https://www.berlin-university-alliance.de/index.html). A.C.H. and S.H. were funded by BMBF (RAPID, https://www.bmbf.de/bmbf/de/home/home_node.html). K.H., M.M., D.F., A.D.G., S.D., S.H., A.C.H. were supported by DFG (SFB-TR 84, A4, A7, B6, Z1A, [https://www.dfg.de)]https://www.dfg.de). We acknowledge financial support from the Open Access Publication Fund of Charité –Universitätsmedizin Berlin and the German Research Foundation (DFG). The funders had and will not have a role in study design, data collection and analysis, decision to publish, or preparation of the manuscript.
