# Supplementary material for: State-of-the-art analytical methods of viral infections in human lung organoids
Source: PLoS One. 2022 Dec 20;17(12):e0276115. doi: 10.1371/journal.pone.0276115 (PMC9767351; doi:10.1371/journal.pone.0276115)
Supplement: S1 File — (PDF) [file pone.0276115.s001.pdf]

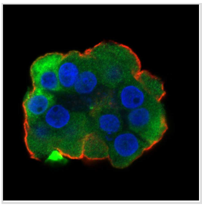

cebataie

# Collection: State-of-the-Art Analytical Methods of Viral Infections in Human Lung Organoids V.(cebataie)

Morris Baumgardt[1], Maren Hülsemann[2], Anna Löwa[1], Diana Fatykhova[1], Karen Hoffmann[1], Mirjana Kessler[3], Maren Mieth[4], Katharina Hellwig[1], Doris Frey[1], Alina Langenhagen[5], Anne Voss[5], Benedikt Obermayer[6], Emanuel Wyler[7], Simon Dökel[5], Achim D. Gruber[5], Ulf Tölch[2], Stefan Hippenstiel[1], Andreas C. Hocke[1], Katja Hönzke[1]

[1]Charité – Universitätsmedizin Berlin, corporate member of Freie Universität Berlin and Humboldt Universität zu Berlin, Department of Infectious Diseases and Respiratory Medicine, Charitéplatz 1, 10117 Berlin, Germany.;

[2]Berlin Institute of Health at Charité (BIH), BIH QUEST Center for Responsible Research, Berlin, Germany;

[3]Charité – Universitätsmedizin Berlin, corporate member of Freie Universität Berlin and Humboldt Universität zu Berlin, Department of Infectious Diseases and Respiratory Medicine, Charitéplatz 1, 10117 Berlin, Germany and Department of Gynecology and Obstetrics, University Hospital, LMU, Munich, Germany;

[4]Department of Infectious Diseases and Respiratory Medicine, Charité - Universitätsmedizin Berlin, corporate member of Freie Universität Berlin, Humboldt-Universität zu Berlin, and Berlin Institute of Health, Berlin, Germany.;

[5]Department of Veterinary Pathology, Freie Universität Berlin, Berlin, Germany;

[6]Berlin Institute of Health at Charité – Universitätsmedizin Berlin, Core Unit Bioinformatics;

[7]Berlin Institute for Medical Systems Biology (BIMSB), Max Delbrück Center for Molecular Medicine in the Helmholtz Association (MDC) and IRI Life Sciences, Institute for Biology, Humboldt Universität zu Berlin, Berlin, Germany.

1 *Works for me*

**Reserved DOI:**
10.17504/protocols.io.5jyl89pw6v2w/v2

morris.baumgardt

DISCLAIMER

Informed written consent was obtained from all volunteers and the study was approved by the Charité Ethics Committee (project 451, EA2/079/13).

ABSTRACT

Organ models have received widespread attention in the study of SARS-CoV-2, the pathogen causing the current COVID-19 pandemic. Human-based organ models can provide strong predictive value to investigate the tropism, virulence, and replication kinetics of viral pathogens.
Applicable to a large set of organoid models and viruses, we provide a step-by-step work instruction for the infection of human alveolar-like organoids with SARS-CoV-2 in this protocol collection. We also prepared a detailed description on state-of-the-art methodologies to assess the infection impact and the analysis of relevant host factors in organoids.
This protocol collection consists of five different sets of protocols. Set 1 describes the protein extraction from human alveolar-like organoids and the determination of protein expression of angiotensin-converting enzyme 2 (ACE2), transmembrane serine protease 2 (TMPRSS2) and FURIN as exemplary host factors of SARS-CoV-2. Set 2 provides detailed guidance on the extraction of RNA from human alveolar-like organoids and the subsequent qPCR to quantify the expression level of e.g., *ACE2* or other host factors of SARS-CoV-2 on RNA level. Protocol set 3 contains an in-depth explanation on how to infect human alveolar-like organoids with SARS-CoV-2 and how to quantify the viral replication by plaque assay and viral E gene-based RT-qPCR. Set 4 provides a step-by-step protocol for the isolation of single cells from infected human alveolar-like organoids for further processing in single-cell RNA sequencing or flow cytometry. Set 5 presents a detailed protocol on how to perform the fixation of human alveolar-like organoids and guides through all steps of immunohistochemistry and *in situ* hybridization to visualize SARS-CoV-2 and its host factors. The infection and all subsequent analytical methods have been successfully validated by biological replications with human alveolar-like organoids based on material from different donors.

COLLECTION INFO

Morris Baumgardt, Maren Hülsemann, Anna Löwa, Diana Fatykhova, Karen Hoffmann, Mirjana Kessler, Maren Mieth, Katharina Hellwig, Doris Frey, Alina Langenhagen, Anne Voss, Benedikt Obermayer, Emanuel Wyler, Simon Dökel, Achim D. Gruber, Ulf Tölch, Stefan Hippenstiel, Andreas C. Hocke, Katja Hönzke . Collection: State-of-the-Art Analytical Methods of Viral Infections in Human Lung Organoids. **protocols.io**
https://protocols.io/view/collection-state-of-the-art-analytical-methods-of-cebataie
Version created by morris.baumgardt

KEYWORDS

Human Lung Organoids, Protein Extraction, Western Blot, RNA Extraction, RT-qPCR, SARS-CoV-2. Infection, Plaque Assay, viral qPCR, Single Cell Isolation, Single Cell RNA Sequencing, Immunohistochemistry, in situ Hybridization

CREATED

Jul 26, 2022

LAST MODIFIED

Aug 22, 2022

COLLECTION INTEGER ID

67650

GUIDELINES

This protocol collection describes the processing of human alveolar-like organoids which have been grown according to Youk et al., 2020. https://doi.org/10.1016/j.stem.2020.10.004.

BEFORE START

Grow the virus stock (SARS-CoV-2 B.1) on Vero E6 cells (RRID:CVCL_0574), please work with maximum passage 3 and sequence the virus stock initially.

SAFETY WARNINGS

SARS-CoV-2 virus and infected material has to be handeled on biosafety level 3 (BSL3).

DISCLAIMER:

Informed written consent was obtained from all volunteers and the study was approved by the Charité Ethics Committee (project 451, EA2/079/13).

ABSTRACT

Organ models have received widespread attention in the study of SARS-CoV-2, the pathogen causing the current COVID-19 pandemic. Human-based organ models can provide strong predictive value to investigate the tropism, virulence, and replication kinetics of viral pathogens.
Applicable to a large set of organoid models and viruses, we provide a step-by-step work instruction for the infection of human alveolar-like organoids with SARS-CoV-2 in this protocol collection. We also prepared a detailed description on state-of-the-art methodologies to assess the infection impact and the analysis of relevant host factors in organoids.
This protocol collection consists of five different sets of protocols. Set 1 describes the protein extraction from human alveolar-like organoids and the determination of protein expression of angiotensin-converting enzyme 2 (ACE2), transmembrane serine protease 2 (TMPRSS2) and FURIN as exemplary host factors of SARS-CoV-2. Set 2 provides detailed guidance on the extraction of RNA from human alveolar-like organoids and the subsequent qPCR to quantify the expression level of e.g., *ACE2* or other host factors of SARS-CoV-2 on RNA level. Protocol set 3 contains an in-depth explanation on how to infect human alveolar-like organoids with SARS-CoV-2 and how to quantify the viral replication by plaque assay and viral E gene-based RT-qPCR. Set 4 provides a step-by-step protocol for the isolation of single cells from infected human alveolar-like organoids for further processing in single-cell RNA sequencing or flow cytometry. Set 5 presents a detailed protocol on how to perform the fixation of human alveolar-like organoids and guides through all steps of immunohistochemistry and *in situ* hybridization to visualize SARS-CoV-2

and its host factors. The infection and all subsequent analytical methods have been successfully validated by biological replications with human alveolar-like organoids based on material from different donors.

FILES

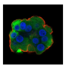

Protein Extraction and Western Blot of Human Lung Organoids
**Version ceattaen**
**by maren.huelsemann**

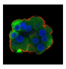

RNA Extraction and RT-qPCR of Human Lung Organoids
**Version ceattaen**
**by morris.baumgardt**

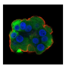

SARS-CoV-2 Infection and Viral Replication of Human Lung Organoids
**Version ceattaen**
**by morris.baumgardt**

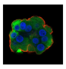

Single Cell Isolation of Human Lung Organoids
**Version ceattaen**
**by morris.baumgardt**

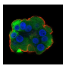

Fixation, Immunohistochemistry and in situ Hybridization of Human Lung Organoids
**Version ceattaen**
**by morris.baumgardt**
