# Supplementary material for: State-of-the-art analytical methods of viral infections in human lung organoids
Source: PLoS One. 2022 Dec 20;17(12):e0276115. doi: 10.1371/journal.pone.0276115 (PMC9767351; doi:10.1371/journal.pone.0276115)
Supplement: S2 File — (PDF) [file pone.0276115.s002.pdf]

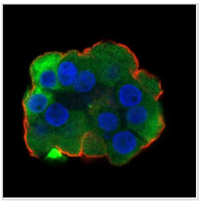

ceastaee

Protein Extraction and Western Blot of Human Lung Organoids V.(ceastaee)

In 1 collection

Morris Baumgardt[1], Maren Hülsemann[2], Diana Fatykhova[1], Stefan Hippenstiel[1], Andreas C. Hocke[1], Katja Hönzke[1]

[1]Charité – Universitätsmedizin Berlin, corporate member of Freie Universität Berlin and Humboldt Universität zu Berlin, Department of Infectious Diseases and Respiratory Medicine, Charitéplatz 1, 10117 Berlin, Germany.;

[2]Berlin Institute of Health at Charité (BIH), BIH QUEST Center for Responsible Research, Berlin, Germany

1 Works for me

Reserved DOI:
10.17504/protocols.io.81wgb6peylpk/v3

maren.huelsemann

DISCLAIMER

Informed written consent was obtained from all volunteers and the study was approved by the Charité Ethics Committee (project 451, EA2/079/13).

ABSTRACT

This protocol describes the protein extraction from human alveolar-like organoids followed by western blotting. It gives a detailed description of the preparation of cell lysate from human alveolar-like organoids, followed by exact steps to perform a western blot and the immunostaining to detect ACE2, TMPRSS2, and FURIN as host factors of SARS-CoV-2.

PROTOCOL INFO

Morris Baumgardt, Maren Hülsemann, Diana Fatykhova, Stefan Hippenstiel, Andreas C. Hocke, Katja Hönzke . Protein Extraction and Western Blot of Human Lung Organoids . **protocols.io**
https://protocols.io/view/protein-extraction-and-western-blot-of-human-lung-ceastaee
Version created by morris.baumgardt

COLLECTIONS

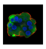

Collection: State-of-the-Art Analytical Methods of Viral Infections in Human Lung Organoids

KEYWORDS

protein extraction, human lung organoids, Sars-CoV-2 hostfactor, ACE-2, Western Blot

CREATED

Jul 26, 2022

LAST MODIFIED

Sep 27, 2022

OWNERSHIP HISTORY

Sep 27, 2022 maren.huelsemann

PROTOCOL INTEGER ID

67634

PARENT PROTOCOLS

Part of collection

Collection: State-of-the-Art Analytical Methods of Viral Infections in Human Lung Organoids

GUIDELINES

This protocol describes the processing of human alveolar-like organoids which have been grown according to Youk et al., 2020. https://doi.org/10.1016/j.stem.2020.10.004.

MATERIALS TEXT

A	B	C
**Substance/Material**	**Company**	**Order Number**
Protein LoBind® Tubes 1.5 mL	Eppendorf	0030108116
Protein LoBind® Tubes 15 mL	Eppendorf	0030122216
7.5% Mini-Protean TGX Precast Gel	Bio Rad	456-1023
Odyssey (PBS) Blocking Buffer	LI-COR	927-40000
DC Assay	Bio Rad	5000116
KIT ECL PRIME DET. REAGENT	Amersham	RPN2232
ECL Western Blotting Substrate	Thermo	32109
cOmplete™, Mini Protease Inhibitor Cocktail	Roche	11836153001
GBX Developer and Fixer	KODAK	

Materials

A	B	C
**Antibody**	**Company**	**RRID**
Anti-β-Actin antibody, Mouse monoclonal, 1:5000 WB, A1978	Sigma	AB_476692
goat-anti-ACE2, 1:100 WB, AF933	R&D	AB_355722
Anti-TMPRSS2, 1:1000, rabbit	abcam	AB_10863728
Anti-FURIN, 1:1000 rabbit	abcam	AB_2801581
mouse anti-goat IgG-HRP	Santa Cruz Biotechnology	AB_628490
mouse anti-rabbit IgG-HRP	Santa Cruz Biotechnology	AB_628497
m-IgGκ BP-HRP anti-mouse	Santa Cruz Biotechnology	AB_2687626

Antibodies

A	B
**Substance**	**Volume**
25 mM Tris (pH 8)	25 mL
137 mM NaCl	27.4 mL
10 % Glycerol	100 mL
0.1 % SDS	1 g
0.5 % Na deoxycholate DOC	5 g
1 % NP40	10 mL
2 mM EDTA (pH 8)	4 mL

RIPA-Buffer (1000 mL) (store at 4°C)

- Dissolve one cOmplete Protease Inhibitor tablet in 2 mL dist. water (store at 2 to 8 °C for 1 to 2 weeks, or at least 12 weeks at -15 to -25 °C)
- Add fresh 40 µL Protease Inhibitor Cocktail 3 to 1 mL RIPA buffer

A	B
**Substance**	**Volume**
10 % SDS	1 g
50 % Glycerol	5 mL
25 % β-Mercaptoethanol	2.5 mL
0.01 % Bromphenol blue	0.001 g
312 mM Tris/HCl pH 6.8	3.1 mL of 1 M solution

SDS-Sample-Buffer (store at -20°C)

A	B
**Substance**	**Volume**
1.5 M Tris pH 9	181.71 g
0.4 % Temed	4 mL
0.4 % SDS	4 g

Running-Buffer (store at 4°C)

- Adjust pH 9 using HCl, adjust to 1 L with dH2O

A	B
**Substance**	**Volume**
0.14 M Tris pH 6.8	16.958 g
0.11 % Temed	1.10 mL
0.11 % SDS	1.10 g

Stacking-Buffer (store at 4°C)

- Adjust pH 6.8 using HCl, adjust to 1 L with dH2O

A	B	C	D
Reagent	Molecular weight	Molarity of 1X	Add for 1 L of 1x
NaCl	58.44	137 mM	8 g
KCl	74.54	2.7 mM	0.2 g
Na2HPO4	141.96	10 mM	1.44 g
KH2PO4	136.08	1.8 mM	0.24 g

1x PBS (store at RT)

- to prepare 0.05% (V/V) **PBS-T** buffer, add 50 µL Tween 20 to 1 L of 1x PBS

10x Blotting-Buffer:

- Dissolve 30.1 g Tris and 144 g Glycin in dH2O
- Adjust to 1 L, store at 4°C
- Before use add 10 % Methanol (1 x Blotting-Buffer = 100 mL 10 x Buffer + 100 mL Methanol + 800 mL dH2O)

10x SDS Page Running-Buffer:

- Dissolve 10 g SDS, 30.3 g Tris and 144.1 g Glycin in 800 mL dH2O
- Adjust to 1 L with dH2O, store at 4°C

DISCLAIMER:

Informed written consent was obtained from all volunteers and the study was approved by the Charité Ethics Committee (project 451, EA2/079/13).

BEFORE STARTING

Please use protein low-bind tubes for all steps.

Prepare cell lysate of alveolar-like organoids 15m

1 You need a sufficient amount of organoids (approx. 300,000 cells per well) and organoids should be collected from minimum two wells of a 24-well plate (total ~600,000 cells).

2 From here all steps **On ice** .

Keep tubes, reagents and buffers on ice.

3 Remove organoid media by aspiration.

4 Add **1 mL** ice-cold PBS to the organoids.

5 **On ice** Transfer organoids to **15 mL** LoBind Tube (with 1000 µL pipette).

6 Centrifuge **300 x g, 4°C, 00:05:00** . 5m

7 **On ice** Remove supernatant carefully.

8 **On ice** Make sure the protease inhibitor is added to the RIPA buffer.

9 Add **75 µL** RIPA buffer + Protease inhibitor to pellet.

10 Breake organoid pellet by repeated resuspension (3 times) using a disposable syringe with 27G needle.

11 Transfer sample to 1.5 mL LoBind tube.

12 Centrifuge **16000 x g, 4°C, 00:10:00** . 10m

13 On ice Collect and transfer supernatant to 1.5 mL LoBind tube (~ 50 µL).

14 Freeze at -80 °C optional or continue.

Protein quantification and preparation before Western blot 7m

15 Use 5 µL of RIPA-lysate in DC Assay (a dilution of the sample might be required, 1:10 or 1:50 in PBS, follow manufacturer's instructions) for protein quantification.

16 Prepare appropriate amount of sample by using 50 µg protein lysate according to DC assay results with 4x Laemmli protein sample buffer for SDS-PAGE.

17 Shake for 00:07:00 at 95 °C (1000 rpm). 7m

SDS-PAGE

18 Commercial gel used: Bio Rad 7.5% (Mini-Protean TGX Precast Gels Cat# 456-1023).

19 Arrange gel chambers inside the electrophorator and fill the tank with 1x Running-Buffer (fill the chamber formed by the gels with new 1x Running-Buffer, check if everything is tight and fill the tank with 1x Running-Buffer until it is half full). Remove the comb carefully.

20 Load 5 µL Kaleidoscope Protein Standard in lane 1 (Precision Plus Protein Kaleidoscope Prestained Protein Standards #1610375).

21 Add 50 µg sample/lane (can be increased to 100 µg sample/lane for low expressed proteins).

22 Run the gel for up to 01:30:00 at 100 V (until the blue front line runs out of the gel). 1h 30m

Blotting

23 After the SDS-Page is done, do not switch off the power (due to diffusing proteins) until

everything is prepared for blotting. It is better to reduce the voltage instead.

24 Before use keep bottle of 1x blotting buffer in the freezer for 01:30:00 . Equilibrate PVDF membrane ([M]0.45 micromolar (µM) methanol for 00:00:15) and transfer it to the 1x blotting buffer. Add sponges and filter papers in 1x blotting buffer. For each gel, you need 2 sponges and 4 filter papers. 1h 30m 15s

25 Carefully open gel chamber and equilibrate gel in 1x blotting buffer for 00:02:00 . 2m

26 Create a transfer sandwich as follows:

- White side of retainer (top)
- Sponge
- 2 filter papers
- PVDF Membrane
- SDS Gel
- 2 filter papers
- Sponge
- Black side of retainer (bottom)

27 Check out that there are no air bubbles between the gel and the membrane. Use a pipette to squeeze out extra liquid and bubbles.

28 Relocate the sandwich to the transfer apparatus. Add ice and fill with 1x Blotting-Buffer until the sandwich is completely covered.

29 Transfer for 01:00:00 at 100 V. 1h

Blocking and antibody incubation 2h 11m

30 Wash membrane with PBS.

31 Block membrane in Odyssey blocking solution for 01:00:00 . 1h

32

Cut membrane for multiple protein detection.

33

Add primary antibody (diluted in Odyssey blocking solution pure) and incubate membrane on a shaker overnight at 4 °C .

34 Wash membrane thrice with 1x PBS-T for 00:05:00 each. 5m

35 Incubate membrane for 01:00:00 at room temperature with secondary antibody solution (in 1x PBS-T with 5% milk). 1h

36 Wash membrane twice with 1x PBS-T for 00:05:00 each. 5m

37 Wash membrane with 1x PBS for 00:05:00 . 5m

38 Prepare ECL solution and incubate on membrane according to manufactures instructions (00:02:00 for Thermo Scientific Kit and 00:05:00 for Amersham Kit). The more sensitive Amersham Kit is used for ACE2 detection, all other proteins are detected using the Thermo Scientific Kit. 7m

39 Expose film until an adequate signal can be determined

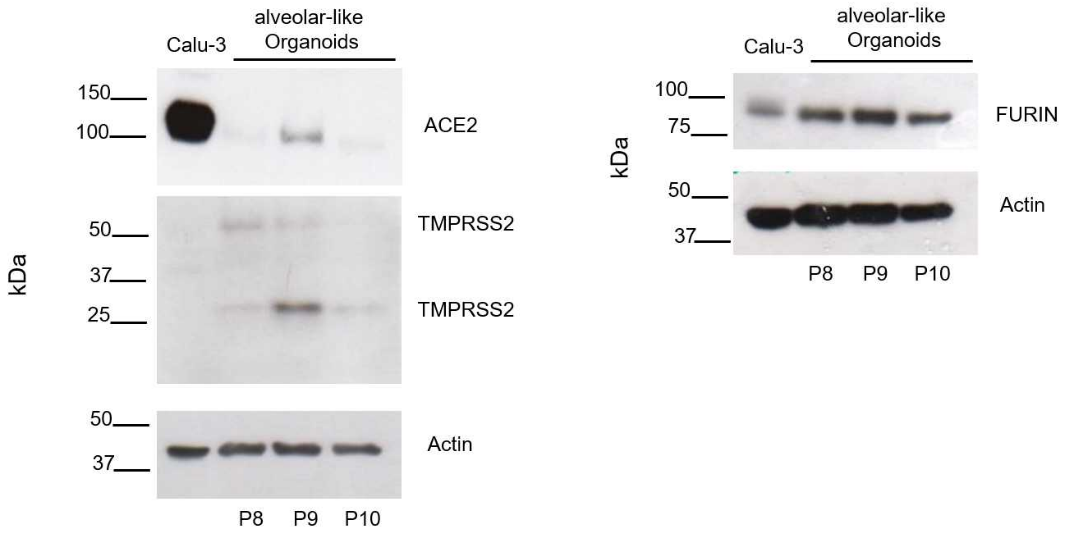

Analysis of constitutive ACE2 (120 kDa), TMPRSS2 (54 and 25 kDa) and FURIN (87 kDa) expression in human alveolar-like organoids by Western blot (three donors).
