# Supplementary material for: State-of-the-art analytical methods of viral infections in human lung organoids
Source: PLoS One. 2022 Dec 20;17(12):e0276115. doi: 10.1371/journal.pone.0276115 (PMC9767351; doi:10.1371/journal.pone.0276115)
Supplement: S3 File — (PDF) [file pone.0276115.s003.pdf]

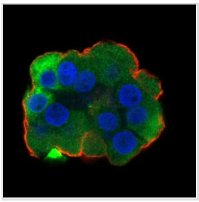

ceaptadn

RNA Extraction and RT-qPCR of Human Lung Organoids V.(ceaptadn)

In 1 collection

Morris Baumgardt[1], Maren Hülsemann[2], Maren Mieth[1], Doris Frey[1], Stefan Hippenstiel[1], Andreas C. Hocke[1], Katja Hönzke[1]

[1]Charité – Universitätsmedizin Berlin, corporate member of Freie Universität Berlin and Humboldt Universität zu Berlin, Department of Infectious Diseases and Respiratory Medicine, Charitéplatz 1, 10117 Berlin, Germany.;

[2]Berlin Institute of Health at Charité (BIH), BIH QUEST Center for Responsible Research, Berlin, Germany.

1 Works for me

Reserved DOI:
10.17504/protocols.io.14egn7z8zv5d/v3

morris.baumgardt

DISCLAIMER

Informed written consent was obtained from all volunteers and the study was approved by the Charité Ethics Committee (project 451, EA2/079/13).

ABSTRACT

This protocol describes the RNA extraction from human alveolar-like organoids followed by the perfomance of a RT-qPCR to quantify the amount of hostfactors (*ACE2*, *TMPRSS2*, and *FURIN*) of SARS-CoV-2. The protocol contains detailed steps for the lysis of human alveolar-like organoids, as wells as the RNA extraction followed by the reverse transcription into cDNA. In the next section the protocol describes in detail how to quantify the gene expression with the TaqMan gene expression kit to obtain donor dependent C_t values.

PROTOCOL INFO

Morris Baumgardt, Maren Hülsemann, Maren Mieth, Doris Frey, Stefan Hippenstiel, Andreas C. Hocke, Katja Hönzke . RNA Extraction and RT-qPCR of Human Lung Organoids . **protocols.io**
https://protocols.io/view/rna-extraction-and-rt-qpcr-of-human-lung-organoids-ceaptadn
Version created by morris.baumgardt

COLLECTIONS

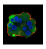

Collection: State-of-the-Art Analytical Methods of Viral Infections in Human Lung Organoids

KEYWORDS

RNA extraction, human lung organoids, TaqMan, RT-qPCR, Sars-CoV-2 hostfactors

CREATED

Jul 26, 2022

LAST MODIFIED

Sep 27, 2022

PROTOCOL INTEGER ID

67631

PARENT PROTOCOLS

Part of collection

Collection: State-of-the-Art Analytical Methods of Viral Infections in Human Lung Organoids

GUIDELINES

This protocol describes the processing of human alveolar-like organoids which have been grown according to Youk et al., 2020. https://doi.org/10.1016/j.stem.2020.10.004.

MATERIALS TEXT

A	B	C	D
**Substance**	**Company**	**Order number**	**Concentration**
GlutaMax 100x	invitrogen	35050-038	5 mL/500 mL medium
Hepes	invitrogen	15630-056	5 mL/500 mL medium
Advanced DMEM/F12	invitrogen	12634-034	1x

Composition of base medium

A	B	C
**Substance**	**Company**	**Order number**
High Capacity cDNA Reverse Transcription Kit	Applied biosciences	4368814
RNeasy Mini Kit (50)	Qiagen	74104
Gene Expression Master Mix	AppliedBiosystems by ThermoFisher Scientific	4369510
Ambion™ Nuclease Free Water	Invitrogen By ThermoFisher Scientific	AM 9937
RLT buffer	Qiagen	7921
Bioanalyzer RNA Analysis	Agilent	G2939BA
Agilent RNA 6000 Nano Kit	Agilent	5067-1511
Biometra TRIO PCR Cycler	analytikjena	846-2-070-720
β-Mercaptoethanol	Merck	M6250

Reagents, kits and devices for RNA extraction and qPCR

A	B	C
TaqMan Gene Expression Assay	ThermoFisher Scientific	4331182
	ACE2	Hs01085333_m1
	TMPRSS2	Hs01122322_m1
	FURIN	Hs00965485_g1
	GAPDH	Hs02758991_g1
	ACTIN	Hs01060665_g1

TaqMan Gene Expression Assay and primers

SAFETY WARNINGS

If working with SARS-CoV-2 infected material please be sure to work at biosafety level 3 (BSL3) until step 7.

DISCLAIMER:

Informed written consent was obtained from all volunteers and the study was approved by the Charité Ethics Committee (project 451, EA2/079/13).

BEFORE STARTING

To avoid contaminations RNAase/DNAase free reagents, consumables and filter tips must be used at all steps.
Please use RNA low bind tubes.

Organoid lysis for RNA isolation

1 Grow your 3D model as described.

> Youk J, Kim T, Evans KV, Jeong YI, Hur Y, Hong SP, Kim JH, Yi K, Kim SY, Na KJ, Bleazard T, Kim HM, Fellows M, Mahbubani KT, Saeb-Parsy K, Kim SY, Kim YT, Koh GY, Choi BS, Ju YS, Lee JH (2020). Three-Dimensional Human Alveolar Stem Cell Culture Models Reveal Infection Response to SARS-CoV-2.. Cell stem cell.
> https://doi.org/10.1016/j.stem.2020.10.004

2 Remove the entire organoid medium from the wells.

3 Add **1 mL** ice-cold base medium and collect Cultrex with organoids in a 2 mL tube and flush well with additional **1 mL** base medium and collect also.

4 To disolve the Cultrex place the tube at **4 °C** for **00:05:00** . 5m

5 Centrifuge at **900 x g, 4°C, 00:05:00** . 5m

6 Carefully remove supernatant and add **350 µL** RLT Puffer + β-Mercaptoethanol to the organoids (pellet).

7 Place supernatant in a 1.5 mL safelock tube.

> ⚠ In case the organoids were infected with a virus they can now be transported out of the biosafety level 3 (BSL3) laboratory

Isolate RNA using „RNeasy Mini Kit” from Qiagen (Cat No./ID 74104).

This step represents the RNA isolation protocol based on the „RNeasy Mini Kit” from Qiagen (Cat No./ID 74104) - no changes have been made to the protocol included in the kit

8 After isolation, measure RNA quality via RIN (RNA Integrity Number) using 2100 Bioanalyzer instrument (manufacturer's instructions, no changes have been made to the protocol included in the kit "Agilent RNA 6000 Nano Kit"). High-quality RNA will contain an RIN of at least 8. All samples used in this protocol had a RIN of 10.

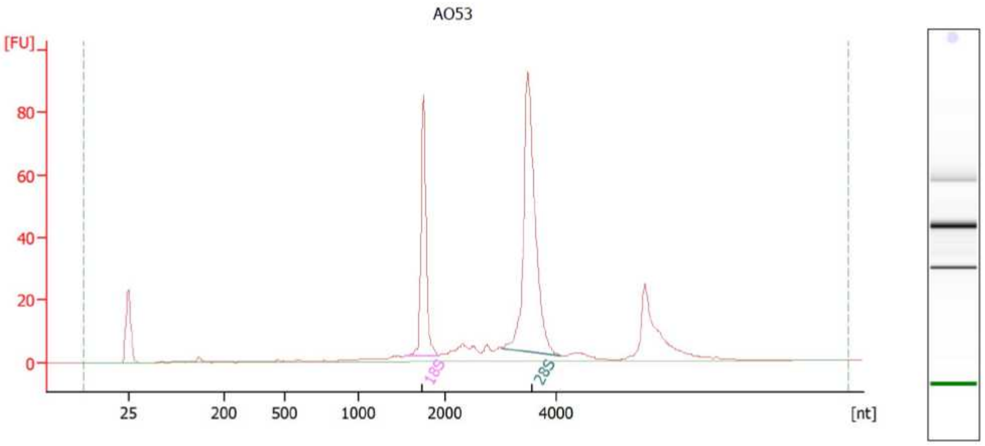

Overall Results for sample 1 : AO53

RNA Area:	392.8	RNA Integrity Number (RIN):	10 (B.02.08)
RNA Concentration:	176 ng/µl	Result Flagging Color:	
rRNA Ratio [28s / 18s]:	2.1	Result Flagging Label:	RIN:10

Fragment table for sample 1 : AO53

Name	Start Size [nt]	End Size [nt]	Area	% of total Area
18S	1,539	1,930	75.0	19.1
28S	3,025	4,076	158.2	40.3

Examplary RNA integrity check using a Bioanalyzer. This RIN example contains a RIN of 10 (completely intact).

Preparation of 2x RT master mix and cDNA reverse transcription 2h 15m

9 ⚠

All steps **On ice** .

10 To quantify the amount of RNA before reverse transcription use **1.5 µL** of your RNA solution using the NanoDrop.

11 Allow the kit components to thaw on ice (high capacity cDNA reverse transcription kit, Applied

biosciences).

12 Calculate the volume of components needed to prepare the required number of reactions.

13 Prepare the RT master mix on ice.

14 One reaction needs:

A	B
**Reagent**	**Amount (µL)**
10x RT Buffer	2
25x dNTP Mix (100mM)	0.8
10x RT Random Primers	2
MultiScribe™ RT	1
Nuclease-free H2O	4.2
TOTAL	10

Master mix (reverse transcription)

15 According to NanoDrop-measurement prepare an RNA/water solution containing **1 µg** RNA/ **10 µL** water in a thin wall PCR tube. If RNA concentration is low, **500 ng** RNA/ **10 µL** water is also sufficient.

16 Include RT control: use water instead of RNA, run one control with each primer.

17 Add **10 µL** of 2x reverse transcription master mix to each tube.

18 Use the following program for reverse transcription:

A	B	C
**Step**	**Temperature**	**Duration**
**1**	25°C	10 min
**2**	37°C	2 h
**3**	85°C	5 min
**4**	4°C	infinite

Program for Reverse Rranscription in a Thermal Cycler

19 Add **80 µL** H_2O to each tube (cDNA).

20 Use the cDNA directly for qPCR or store at **-20 °C** .

TaqMan - quantitative PCR 14m 15s

21 Prepare the qPCR master mix. Calculate the volume of components needed to prepare the required number of reactions for each Gene expression assay.

A	B
**Reagent**	**Amount (µL)**
Gene Expression Master Mix	10
H2O	4
TaQMan Gene Expression Assay	1
**Total**	**15**

TaqMan Gene Expression Assay Master Mix (1 Reaction)

22 Perform qPCR, pipetting **15 µL** of respective TaqMan gene expression assay master mix to the bottom of the well, then pipet **5 µL** of cDNA on the upper wall of the well.

23 Seal the plate with a Clear Adhesive Film.

24 Centrifuge the plate for **1200 x g, 00:00:30** . 30s

25 ⚠

Important quality controls:
Run each sample in duplicates.
Always run house keeping genes (e.g., *GAPDH* and *ß-ACTIN*) and gene of interest on the same plate.
Include water control for each primer.
Include RT control from previous step.

26 Run the qPCR reaction in a Thermal Cycler:

A	B	C	D
**Stage**	**Temperature**	**Duration**	**Repetitions**
**1**	50°C	2 min	1
**2**	95°C	10 min	1
**3**	95°C	15 s	40
**4**	60°C	1 min	40

qPCR Program (PCR Volume = 20 µL)

Analysis

27 **Exemplary result:**

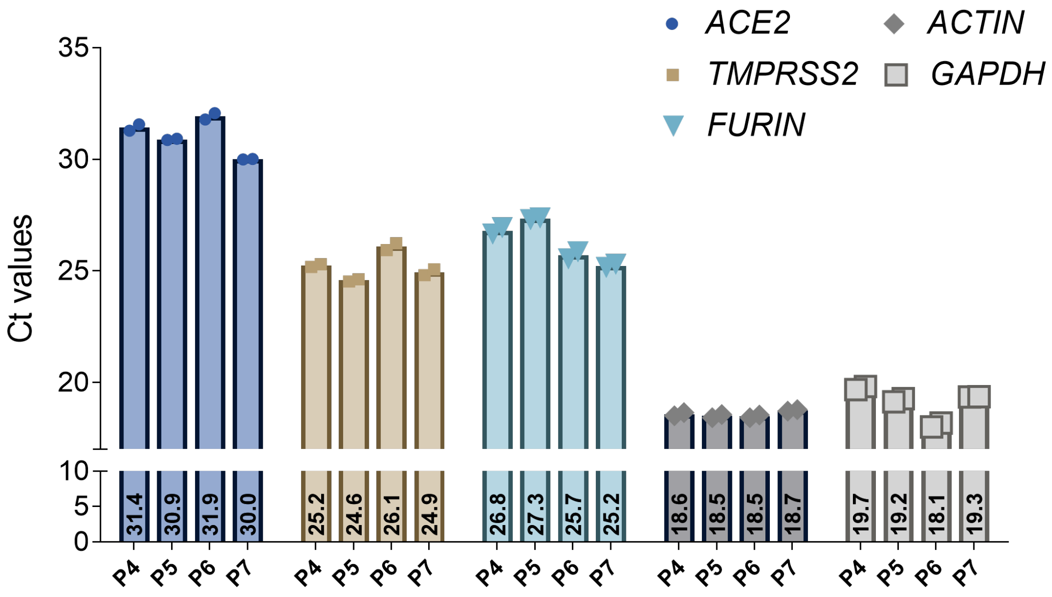

Examplary mRNA expression (Ct values) of *ACE2*, *TMPRSS2*, and *FURIN* in human alveolar-like organoids (*GAPDH* and *ß-ACTIN* serve as reference genes, 4 donors)
