# Supplementary material for: State-of-the-art analytical methods of viral infections in human lung organoids
Source: PLoS One. 2022 Dec 20;17(12):e0276115. doi: 10.1371/journal.pone.0276115 (PMC9767351; doi:10.1371/journal.pone.0276115)
Supplement: S4 File — (PDF) [file pone.0276115.s004.pdf]

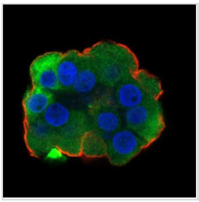

ceaqtadw

SARS-CoV-2 Infection and Viral Replication of Human Lung Organoids V. (ceaqtadw)

In 1 collection

Morris Baumgardt[1], Maren Hülsemann[2], Karen Hoffmann[1], Mirjana Kessler[3], Stefan Hippenstiel[1], Andreas C. Hocke[1], Katja Hönzke[1]

[1]Charité – Universitätsmedizin Berlin, corporate member of Freie Universität Berlin and Humboldt Universität zu Berlin, Department of Infectious Diseases and Respiratory Medicine, Charitéplatz 1, 10117 Berlin, Germany.;

[2]Berlin Institute of Health at Charité (BIH), BIH QUEST Center for Responsible Research, Berlin, Germany;

[3]Charité – Universitätsmedizin Berlin, corporate member of Freie Universität Berlin and Humboldt Universität zu Berlin, Department of Infectious Diseases and Respiratory Medicine, Charitéplatz 1, 10117 Berlin, Germany and Department of Gynecology and Obstetrics, University Hospital, LMU, Munich, Germany

1 *Works for me*

Reserved DOI:
10.17504/protocols.io.j8nlkkepxl5r/v3

morris.baumgardt

DISCLAIMER

Informed written consent was obtained from all volunteers and the study was approved by the Charité Ethics Committee (project 451, EA2/079/13).

ABSTRACT

This protocol describes the working steps to infect human alveolar-like organoids with SARS-CoV-2 and quantify the viral replication at defined time points post infection via plaque assay and viral E gene quantitative reverse transcription PCR (RT-qPCR).

PROTOCOL INFO

Morris Baumgardt, Maren Hülsemann, Karen Hoffmann, Mirjana Kessler, Stefan Hippenstiel, Andreas C. Hocke, Katja Hönzke . SARS-CoV-2 Infection and Viral Replication of Human Lung Organoids. **protocols.io**
https://protocols.io/view/sars-cov-2-infection-and-viral-replication-of-huma-ceaqtadw
Version created by morris.baumgardt

COLLECTIONS

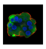

Collection: State-of-the-Art Analytical Methods of Viral Infections in Human Lung Organoids

KEYWORDS

infection, Plaque Assay, viral qPCR, Sars-CoV-2, human lung organoids

CREATED

Jul 26, 2022

LAST MODIFIED

Sep 13, 2022

PROTOCOL INTEGER ID

67632

PARENT PROTOCOLS

Part of collection

Collection: State-of-the-Art Analytical Methods of Viral Infections in Human Lung Organoids

GUIDELINES

This protocol describes the processing of human alveolar-like organoids which have been grown according to Youk et al., 2020. https://doi.org/10.1016/j.stem.2020.10.004

MATERIALS TEXT

For virus stock production and plaque assay Vero E6 cells are required (RRID:CVCL_0574).

A	B	C	D
**Substance**	**Company**	**Order number**	**Concentration**
R-Spondin 1 (cond. med.)	-	-	10%
FGF 7	Peprotech	100-19-100	0.025 µg/mL
FGF 10	Peprotech	100-26-50	0.1 µg/mL
Noggin	Peprotech	120-10C-100	0.1 µg/mL
A83-01	Merck	616454	1 µM
Y-27632	Tocris	1254/10	0.005 mM
SB202190	Sigma	S7067	0.0005 mM
B27 supplement	Invitrogen	17504044	1x
N-Acetylcysteine	Sigma	A9165-5G	1.25 mM
Nicotinamide	Sigma	N0636	0.005 M
Primocin	Invivogen	ant-pm-1	100 U/mL
CHIR	Sigma	SML1046	3 µM

Composition of organoid medium

A	B	C	D
**Substance**	**Company**	**Order number**	**Concentration**
GlutaMax 100x	invitrogen	35050-038	5 mL/500 mL
Hepes	invitrogen	15630-056	5 mL/500 mL
Advanced DMEM/F12	invitrogen	12634-034	1x

Composition of base medium

A	B	C
**Substance/Material**	**Company**	**Order number**
"Sterican® Gr. 20, G 27 x 3/4"" / ø 0,40 x 20 mm, grau"	Braun	4657705
"Omnifix®-F Spritzen, fein dosierbar"	Braun	9161406V
Matrigel (Cultrex)	R&D	3533-005-02
DPBS (1x)	Gibco	14190144
24-well cell culture plate	TPP	TP92424
Disposable Counting Chamber	NanoEntek	DHC-N01

Materials for infection

A	B	C
**Substance/Device**	**Company**	**Order number**
500 mL DMEM, high glucose	Gibco	41965062
Sodium Pyruvate (100 mM)	Gibco	11360070
MEM non-essential amino acid solution (NE:AA, 100x)	Gibco	11140050
Gibco™ OptiPRO™ SFM	Gibco	11530426
Gelatine	Sigma	48723-500G-F
Avicel	FMC Health and Nutrition	RC-591
DMEM, w: 4.5 g/L Glucose, w: L-Glutamine, w/o: Sodium pyruvate, w/o: NaHCO3, Powder	PAN Biotech	P03-0710
37% Formaldehyde	Roth	7398,4
crystal violet	Roth	T123.1
Cooling rack ROTILABO®	Roth	EP69.1
Vortex 3	IKA	IK0003340000

Materials for plaque assay

A	B
**Substance**	**Preparation**
Vero E6 Medium	500 mL DMEM (1x) + 10 % FCS + 1 % NE:AA + 1 % sodium pyruvate
Gelatine medium	5% gelatine in water, autoclave, dilute 1:10 in OptiPRO medium (Gibco)
Avicel 2,4%	700 mL H20 + 16,8 g Avicel powder, stir over night and autoclave
2xDMEM	500 mL DMEM (1x) +6,77 g DMEM powder (with 1.5 g/L D-Glucose, without Na-Pyruvat, with L-Glutamine, without NaHCO3), +100 mL FCS, +10 mL NE:AA, +10 mL sodium pyruvate (100 mM) and sterile filtrate
Crystal violet stock solution	10 g Crystal violet, 50 mL Formaldehyde 37%, 100 mL Ethanol (99.9%), 350 mL deionized water
Crystal violet working solution	100 mL Crystal violet stock solution, 100 mL Formaldehyde (37%), 800 mL deionized water

Medium preparation for plaque assay

A	B	C	D
**Substance/Material**	**Company**	**Order number**	**Sequence**
Thermomixer comfort	Eppendorf	p1844	
NucleoSpin RNA Virus Kit	Macherey-Nagel	740956.250	
SuperScript™ III One-Step RT-PCR System with Platinum™ Taq DNA Polymerase	Thermo Fisher	12574018	
E_Sarbeco_F			ACAGGTACGTTAATAGTTAATAGCGT
E_Sarbeco_R			ATATTGCAGCAGTACGCACACA
E_Sarbeco_Probe			FAM-ACACTAGCCATCCTTACTGCGCTTCG-BBQ
PCR grade H2O, LightCycler® Probes Master	Roche	04 887 301 001	
LightCycler® 480 System	Roche		

Materials for viral quantitative reverse transcription PCR (E gene assay)

SAFETY WARNINGS

SARS-CoV-2 virus and infected material has to be handeled on biosafety level 3 (BSL3).

DISCLAIMER:

Informed written consent was obtained from all volunteers and the study was approved by the Charité Ethics Committee (project 451, EA2/079/13).

BEFORE STARTING

Grow the virus stock (SARS-CoV-2 B.1) on Vero E6 cells (RRID:CVCL_0574), please work with maximum passage 3 and sequence the virus stock initially.

One day before the plaque assay will be performed, Vero E6 cells need to be seeded in order to ensure confluency and readiness for the plaque assay the next day. Therefore, 175,000 cells per 24-well are seeded in 500 µL Vero E6 medium. The next day, verification is made to ensure confluency of the cells.

Cultivation of human alveolar-like organoids

1 Grow your 3D model as described

Youk J, Kim T, Evans KV, Jeong YI, Hur Y, Hong SP, Kim JH, Yi K, Kim SY, Na KJ, Bleazard T, Kim HM, Fellows M, Mahbubani KT, Saeb-Parsy K, Kim SY, Kim YT, Koh GY, Choi BS, Ju YS, Lee JH (2020). Three-Dimensional Human Alveolar Stem Cell Culture Models Reveal Infection Response to SARS-CoV-2.. Cell stem cell. https://doi.org/10.1016/j.stem.2020.10.004

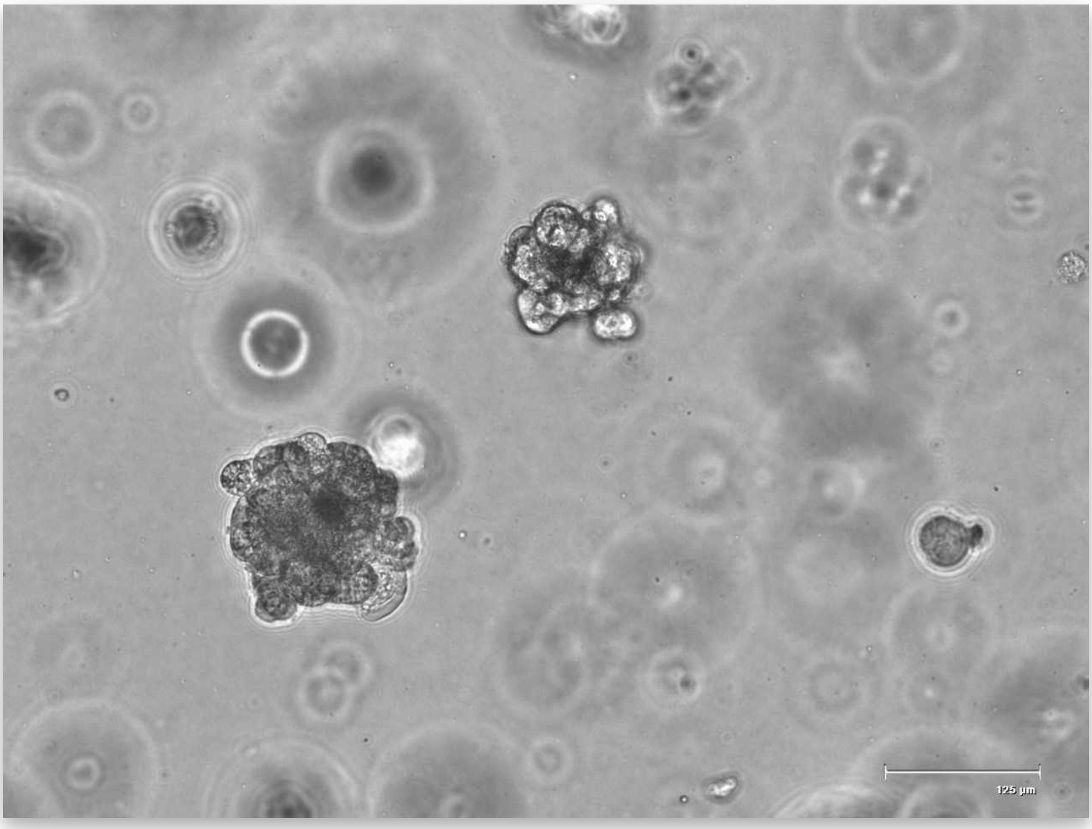

Differentiated Alveolar Organoids (scale bar = 125 µm)

Cell counting of human alveolar-like organoids

2 A specific cell number is needed to calculate the virus MOI (multiplicity of infection).

Carefully remove organoid medium of one well.

3 Add 1 mL (4 °C) cold base medium and collect Cultrex with organoids in a 15 mL tube, flush well with additional 1 mL base medium.

4 Centrifuge at 300 x g, 4°C, 00:05:00 and carefully remove supernatant. 5m

5 Resuspend pellet in 1 mL TrypLE Express for 00:15:00 in a shaking wather bath (37 °C) and add 1 mL base medium afterwards. 15m

6 Produce a single cell solution by resuspending three times with a disposable syringe with needle (27G).

7 Count single cells using a disposable Counting Chamber (Neubauer improved).

Infection of human alveolar-like organoids 1h 50m

8 Carefully remove organoid medium.

9 Add 1 mL (4 °C) cold base medium and collect the suspension containing Cultrex and organoids in a 15 mL tube, flush well with additional 1 mL cold base medium.

10 Centrifuge at 300 x g, 4°C, 00:05:00 . 5m

11 Carefully remove supernatant.

12 Resuspend pellet in 1 mL cold base medium.

13 Break up the organoids by repeated (2-3x) resuspension using a disposable syringe with needle (27G).

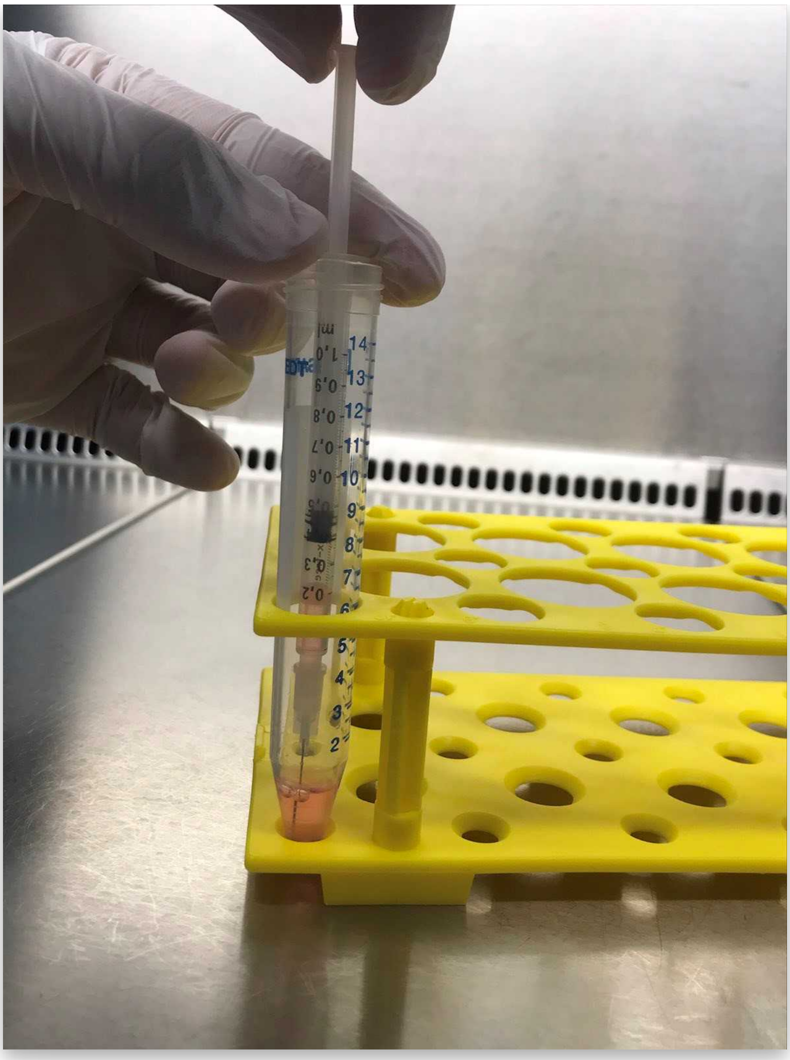

How to break up the organoids with a disposable syringe in a 15 mL tube

14 Pipette virus solution (stock) to the organoids (MOI = 1), mix by pipetting (1 mL total volume per 15 mL tube) and incubate 01:00:00 at 37 °C . Shake the tube gently every 15 mins. 1h

MOI calculation:

(Cell number per well x MOI)/virus stock titer = µL virus solution to be added per well.

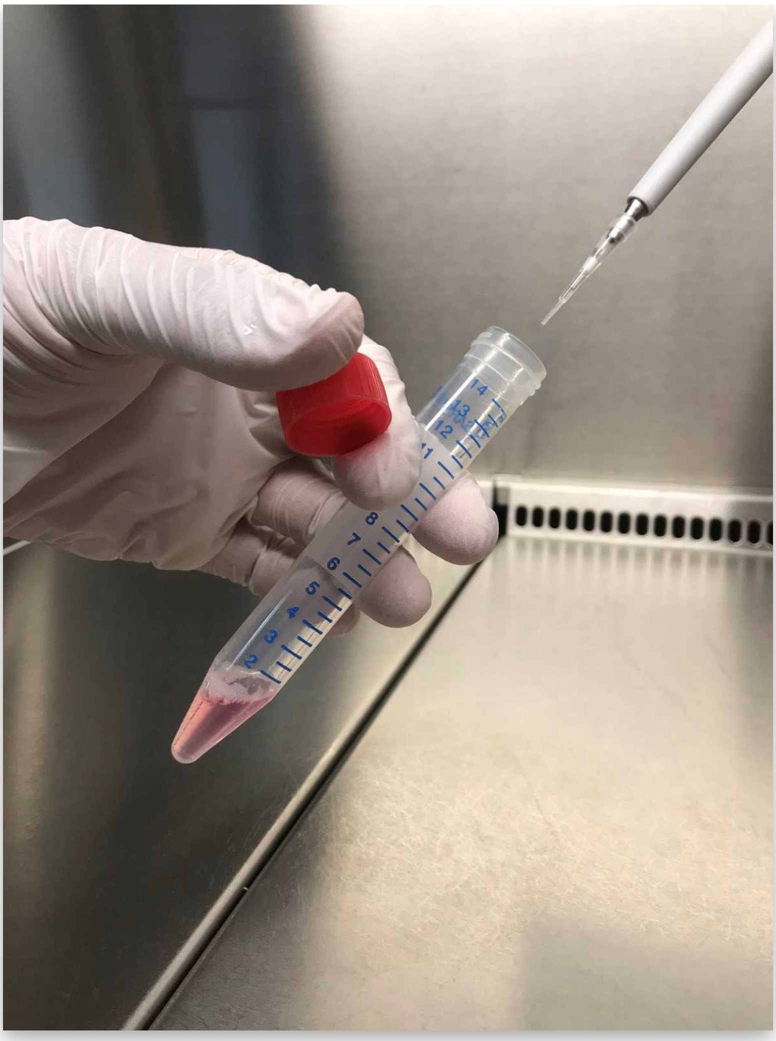

How to add the viral solution to the organoids

15 Centrifuge at 300 x g, 4°C, 00:05:00 . 5m

16 Transfer 50 µL from the supernatant for input titer control (for **viral qPCR** later) in a new 1.5 mL tube and freeze at -80 °C at BSL3 (biosafety level 3) until further processing.

17 Take 100 µL from the supernatant for input titer control (for **Plaque Assay** later) and mix 100 µL supernatant with 100 µL gelatin medium in a new 1.5 mL tube and freeze at -80 °C at BSL3 (biosafety level 3) until further processing in the plaque assay.

18 Carefully remove and discard as much remaining supernatant as possible from the original tube so that only oganoids remain.

19 Wash organoids with 500 µL PBS (1x).

20 Centrifuge at 300 x g, 4°C, 00:05:00 . 5m

21 Place tube on ice, resuspend pellet in thawed Cultrex (50 µL and 4 °C) and add into one well of one pre-warmed 24-well plate (37 °C).

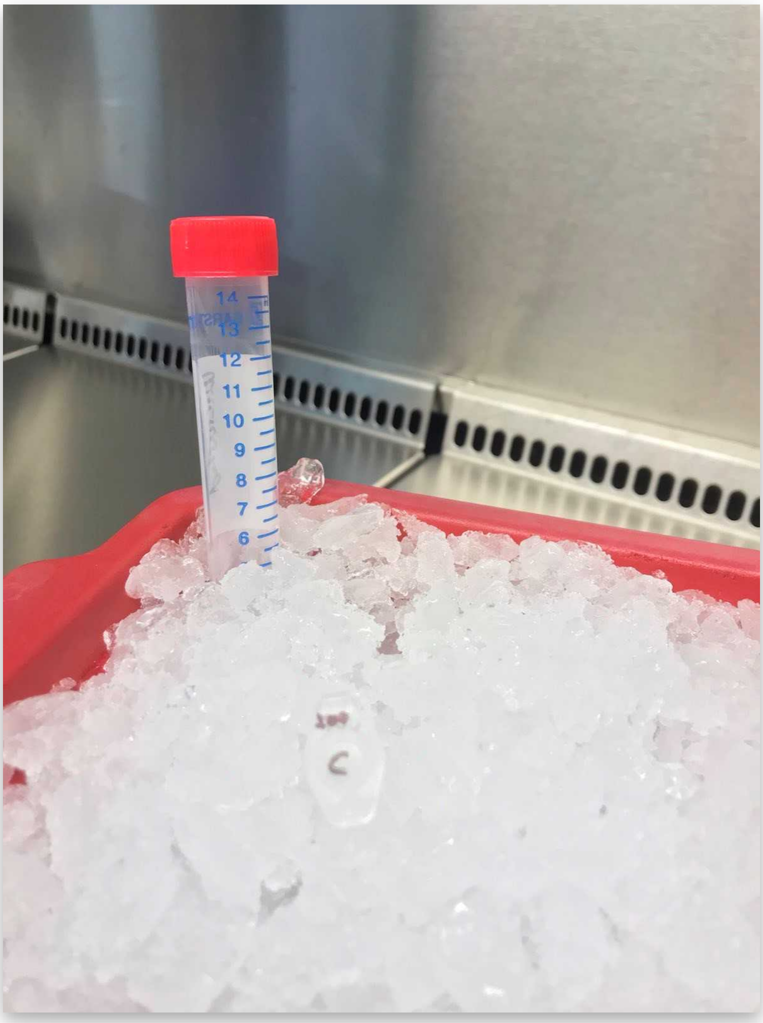

Tube with organoids and Cultrex are placed on ice

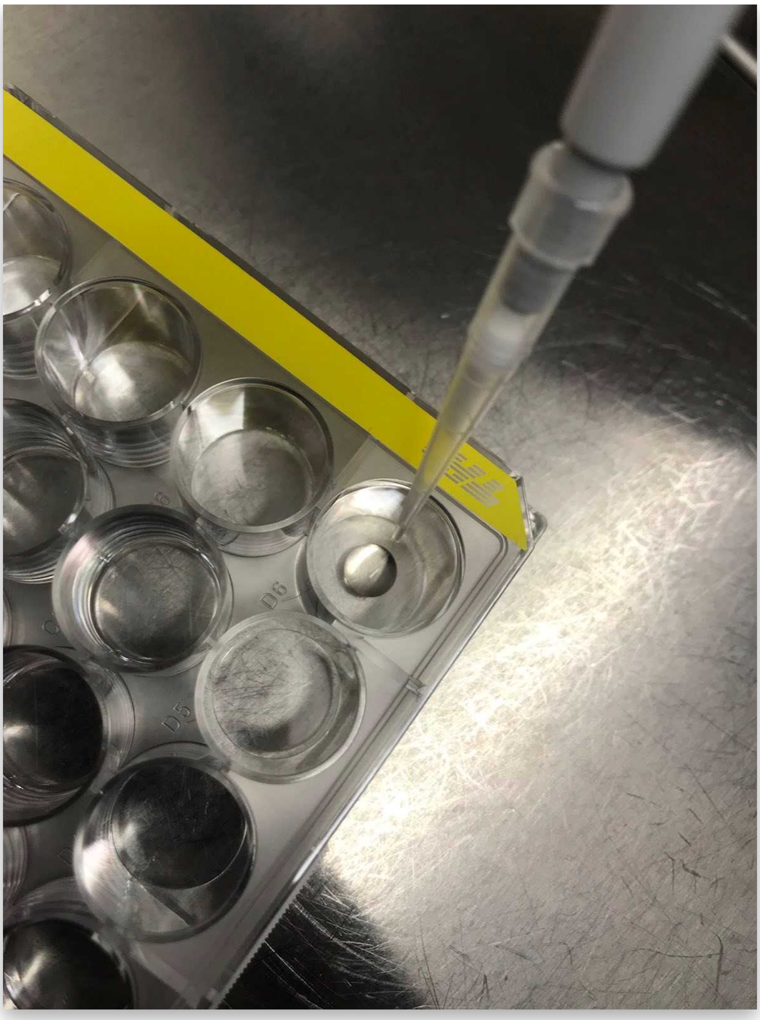

One organoid containing Cultrex drop should be placed in the centre of a pre warmed 24-well plate

22 Incubate **00:30:00** at **37 °C** . 30m

23 Add **500 µL** organoid medium and incubate at **37 °C and 5% CO_2** .

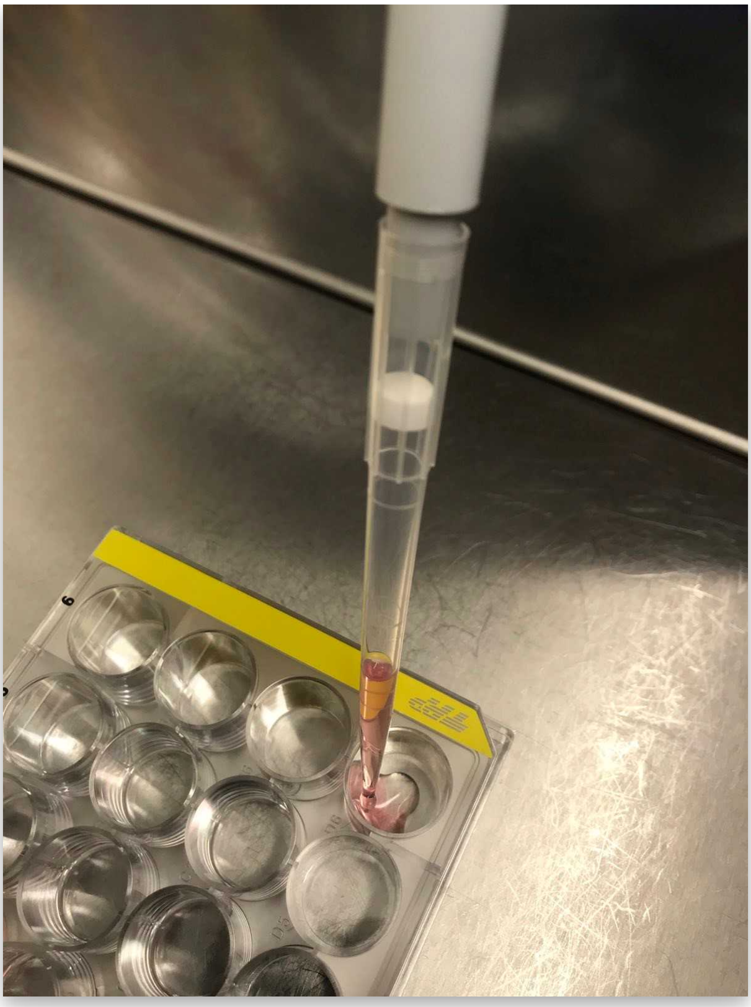

After solidification of Cultrex the medium is added

Plaque Assay 3d 1h 45m

24 Samples for plaque titration are taken from the organoid culture supernatant at different time points/hours post infection (hpi) as described here:

25 Pipette Cultrex and medium vigorously up and down (using 1000 µL pipette) in the well until the Cultrex is homogeneously dispersed in the medium and transfer Cultrex, organoids and virus containing medium into a **1.5 mL** tube.

26 For the complete dissolution of the Cultrex and thereby remove all organoids and viral particles from the matrix, cool the tube with organoid mixture for **00:10:00** at **4 °C** using a pre-cooled cooling rack. 10m

27 After **00:10:00** at **4 °C** centrifuge at **300 x g, 4°C, 00:05:00** . 15m

28 Take 100 µL from the supernatant at the desired time points (e.g. input (0 h), 1 h, 24 h, 48 h, 72 h, 96 h) and mix 100 µL supernatant with 100 µL gelatin medium in a new 1.5 mL tube and freeze at -80 °C at BSL3 (biosafety level 3) until further processing. At the same time, freeze 50 µL of pure supernatant (without gelatin) in another 1.5 mL tube, as these will be used later for viral RT-qPCR (-80 °C).

29 ⚠

Make sure, that Vero E6 cells are confluent before infection (see "Before start").

30 Thaw the obtained supernatants of infected organoids from step 28 and prepare the following dilution serie:

31 Prepare 10-fold serial dilutions: 5s
Mix 50 µL of thawed supernatant and 450 µL OptiPro in a 1.5 mL tube by vortexing (closed cap, moderate power, ~ 00:00:05) to receive a dilution of 10^{-1}. Mix again 50 µL of the 10^{-1} dilution with 450 µL OptiPro in a new tube to receive a 10^{-2} dilution and continue this procedure until the desired dilution is achieved. (In the here described condition for infection a supernatant dilution of not exceeding 10^{-5} is adequate for the detection of SARS-CoV-2 replication in alveolar-like organoids).

32 Infect the Vero E6 cells: (add 200 µL /24-well in duplicates using the desired dilutions).

Exemplary dilutions which can be used:

Input (necessary dilution depends on the virus titer): 10^{-1}, 10^{-2}, 10^{-3}
1 hpi: 10^{-1}, 10^{-2}, 10^{-3}, 10^{-4}
24 hpi: 10^{-2}, 10^{-3}, 10^{-4}, 10^{-5}
48 hpi: 10^{-2}, 10^{-3}, 10^{-4}, 10^{-5}
72 hpi: 10^{-1}, 10^{-2}, 10^{-3}, 10^{-4}
96 hpi: 10^{-1}, 10^{-2}, 10^{-3}, 10^{-4}

33 Incubate the plate at 37 °C for 01:00:00 . 1h

34 Remove supernatant and wash the wells once with PBS.

35 Mix 2.4% Avicel and 2xDMEM in a ratio of 1:1 and add 500 µL Avicel overlay to each well.

36 Incubate at 37 °C for 72:00:00 . 3d

37 Remove Avicel overlay and wash wells once with PBS.

38 Inactivate the entire 24-well plate by adding 6% PFA for 00:30:00 in a biotainer and then export from the BSL3. 30m

39 Remove plates from the biotainer and led them air dry.

40 Add crystal violet working solution and incubate for 00:15:00 . 15m

41 Remove crystal violet working solution.

42 Dry plate under the hood and count plaques.

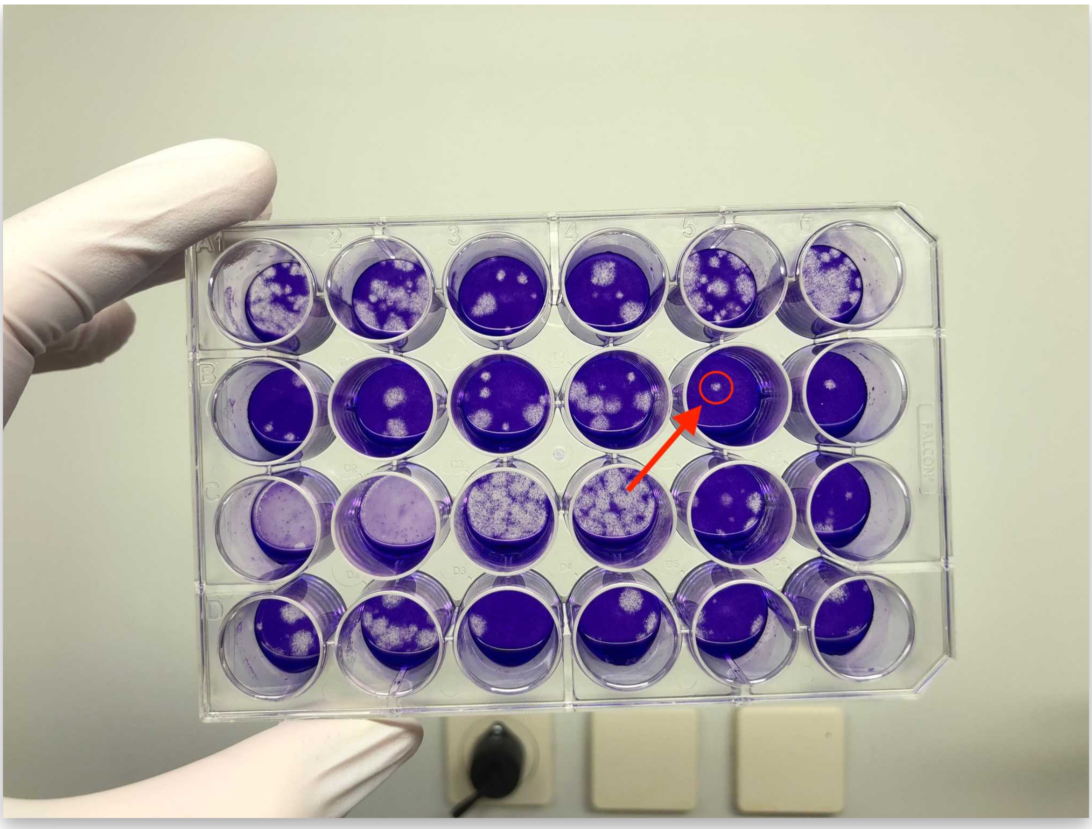

Exemplary Plaque Assay Result (Arrow Shows One Plaque)

43 Document number of plaques in a table and take a picture of the stained plate.

44 Calculate PFU/mL (plaques from two dilution levels and two replicates are counted).

1. Sum of plaques given by the identified virus dilutions = Sum of plaques from 4 wells (e.g. 10^{-3} = 2 wells, 10^{-4} = 2 wells).
2. Titer of SARS-CoV-2 (in PFU/mL) = Number of plaques given by the identified virus dilutions ÷ (lowest dilution factor × 0.44 mL). Calculation is done with the to the lowest dilution referred inoculum volume of 0.44 mL * lowest dilution level.

For example:
10^{-3} dilution: 34 and 37 plaques/well = 71 plaques/two wells
10^{-4} dilution: 1 and 4 plaques/well = 5 plaques/two wells

Sum of all counted plaques / total volume of virus

--> 76 PFU/0.44 mL * 10^3 = 173*10^3 = 1.73*10^5 PFU/mL (the factor 10^3 corresponds to the lowest dilution level that was counted).

45

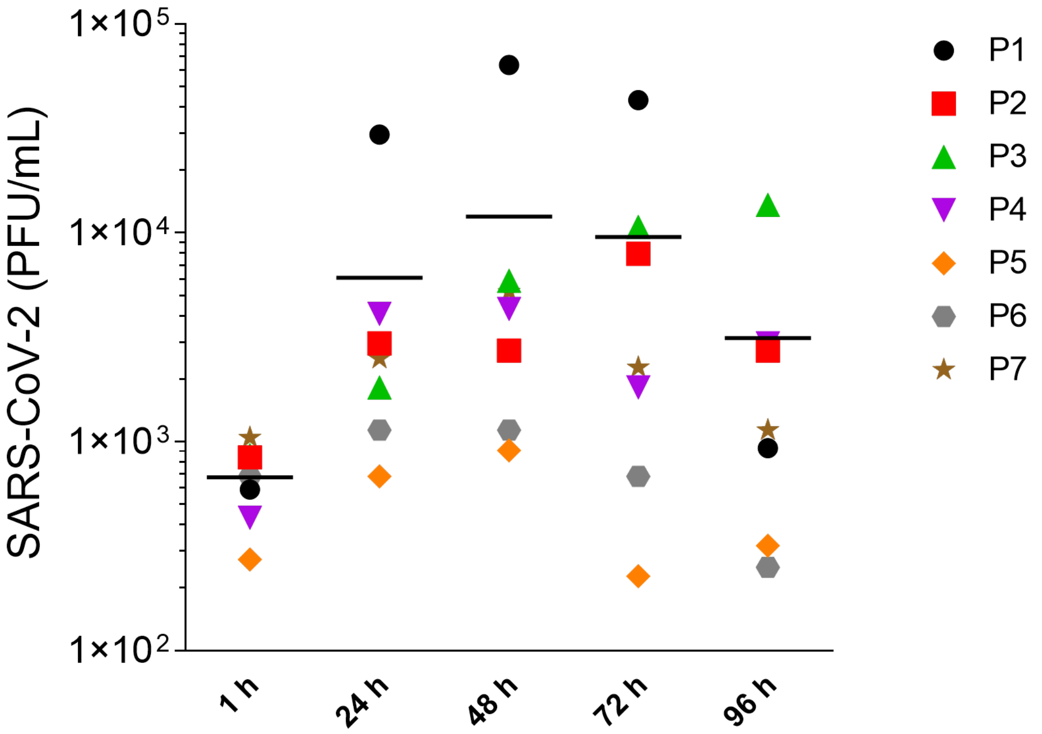

Exemplary replication kinetics (plaque assay) of SARS-CoV-2 infected alveolar-like organoids (7 donors, mean is visualized by black lines).

viral RT-qPCR 10m

46 Samples for the quantification of SARS-CoV-2 RNA in supernatants of infected organoids were taken as described in **step 28**. The samples were taken at different timepoints after infection and frozen.

47 Thaw the frozen **50 µL** supernatant samples at RT.

48 Pipet **50 µL** of virus containing supernatant into **300 µL** of RAV1 buffer with Carrier RNA (as instructed by manufacturer) and vortex.

49 Heat-inactivate the tubes at **70 °C** for **00:10:00** in a thermomixer (**600 rpm**) and export from BSL3. 10m

50 Freeze the samples at **-80 °C** or directly process them for qPCR by first isolating the viral RNA using Nucleospin RNA Virus Kit according to the manufacturer’s protocol.

51 Quantify the viral RNA copies from your samples (**viral qPCR Master Mix table: template**

RNA) using Platinum SuperScript lll One-Step RT-PCR system with Platinum Taq DNA polymerase kit by preparing the master mix shown in the table below.

A	B	C
	**Concentration**	**µL**
PCR-grade H2O		1.8
2X Reaction Mix		6.25
MgSO4	50 mM	0.2
E_Sarbeco_F	10 µM	0.5
E_Sarbeco_R	10 µM	0.5
E_Sarbeco_Probe	10 µM	0.25
SSIII/P.Taq enzyme Mix		0.5
**Total Volume Master Mix**		**10**
**Template RNA**		**2.5**
**Total**		**12.5**

viral qPCR Master Mix

52 Standards for E gene-based quantitative reverse transcription PCR: SARS-CoV-2 E gene ($1x10^5$ copies/µL) as used for clinical diagnostics. In vitro-transcribed control RNA for the E gene assay can be acquired through the European Virus Archive platform (www.european-virus-archive.com).

53 Prepare 10^4, 10^3 and 10^2 copies/µL from the standard stock using nuclease-free water and use four dilutions (10^5-10^2 copies/µL) for qPCR analysis. Caution! avoid contamination of test reaction with the highly concentrated standards.

54 Negative control: PCR-grade, nuclease-free H2O.

55 Evaluation is performed automatically using the Roche LightCycler Software. 13m 45s

A	B	C	D
**Stage**	**Temperature**	**Duration**	**Repetitions**
**1**	55°C	10 min	1
**2**	95°C	3 min	1
**3**	95°C	15 s	45
**4**	58°C	30 s	45

Thermal cycling qPCR program

Corman, V.M.; Landt, O.; Kaiser, M.; Molenkamp, R.; Meijer, A.; Chu, D.K.; Bleicker, T.; Brünink, S.; Schneider, J.; Schmidt, M.L.; et al.. Detection of 2019 novel coronavirus (2019-nCoV) by real-time RT-PCR.. Eurosurveillance.
https://www.eurosurveillance.org/content/10.2807/1560-7917.ES.2020.25.3.2000045

56

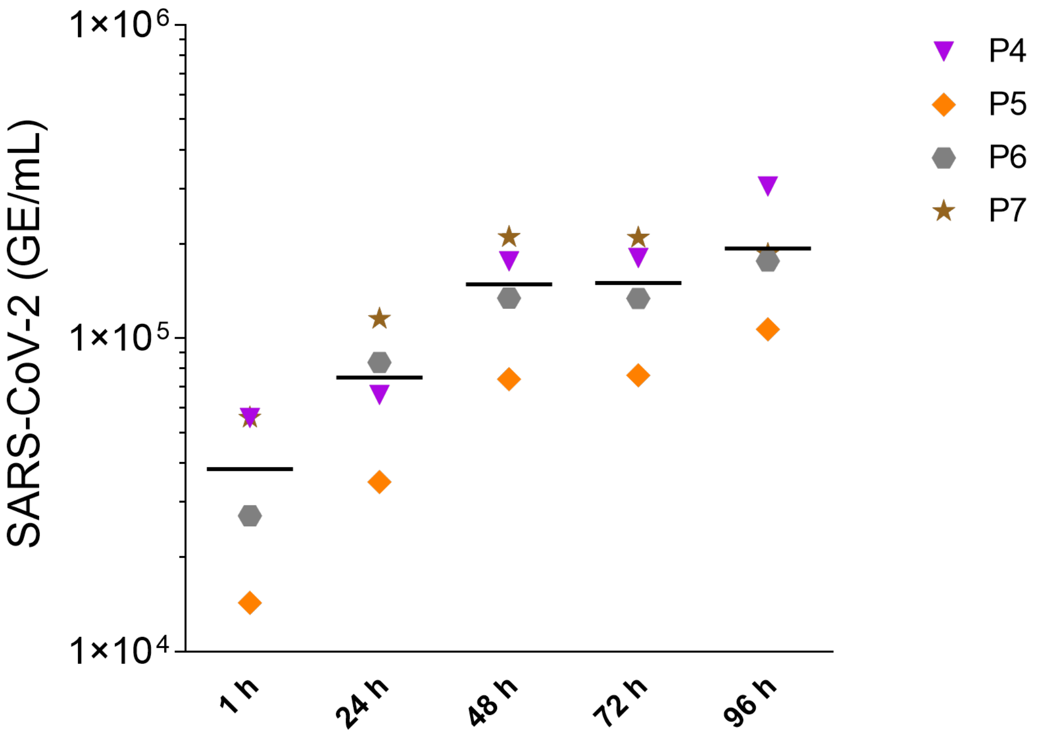

Exemplary replication kinetics (GE; genomic equivalents) of SARS-CoV-2 infected alveolar-like organoids (4 donors, mean is visualized by black lines).
