# Supplementary material for: State-of-the-art analytical methods of viral infections in human lung organoids
Source: PLoS One. 2022 Dec 20;17(12):e0276115. doi: 10.1371/journal.pone.0276115 (PMC9767351; doi:10.1371/journal.pone.0276115)
Supplement: S5 File — (PDF) [file pone.0276115.s005.pdf]

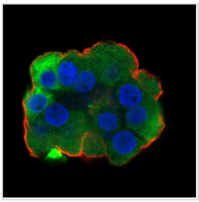

ceartad6

Single Cell Isolation of Human Lung Organoids V.(ceartad6)

In 1 collection

Morris Baumgardt[1], Maren Hülsemann[2], Anna Löwa[1], Benedikt Obermayer[3], Emanuel Wyler[4], Stefan Hippenstiel[1], Andreas C. Hocke[1], Katja Hönzke[1]

[1]Charité – Universitätsmedizin Berlin, corporate member of Freie Universität Berlin and Humboldt Universität zu Berlin, Department of Infectious Diseases and Respiratory Medicine, Charitéplatz 1, 10117 Berlin, Germany.;

[2]Berlin Institute of Health at Charité (BIH), BIH QUEST Center for Responsible Research, Anna-Louisa Karsch Strasse 2, Berlin, Germany.;

[3]Berlin Institute of Health at Charité – Universitätsmedizin Berlin, Core Unit Bioinformatics, Berlin, Germany.;

[4]Berlin Institute for Medical Systems Biology (BIMSB), Max Delbrück Center for Molecular Medicine in the Helmholtz Association (MDC) and IRI Life Sciences, Institute for Biology, Humboldt Universität zu Berlin, Berlin, Germany.

1 *Works for me*

Reserved DOI:
10.17504/protocols.io.3byl4b7orvo5/v3

morris.baumgardt

DISCLAIMER

Informed written consent was obtained from all volunteers and the study was approved by the Charité Ethics Committee (project 451, EA2/079/13).

ABSTRACT

This protocol describes the single cell isolation of infected human alveolar-like organoids in order to perform single cell RNA sequencing or flow cytometry. The protocol focussed on the detailed preparation of samples to prepare single cells for further processing according to the protocols from 10x Genomics for single cell sequencing. The described steps also include the optional fixation of single cells with methanol. In that case, the protocol describes how to rehydrate single cell after methanol fixation to process them for single cell sequencing.

PROTOCOL INFO

Morris Baumgardt, Maren Hülsemann, Anna Löwa, Benedikt Obermayer, Emanuel Wyler, Stefan Hippenstiel, Andreas C. Hocke, Katja Hönzke . Single Cell Isolation of Human Lung Organoids. **protocols.io**
https://protocols.io/view/single-cell-isolation-of-human-lung-organoids-ceartad6
Version created by morris.baumgardt

COLLECTIONS

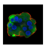

Collection: State-of-the-Art Analytical Methods of Viral Infections in Human Lung Organoids

KEYWORDS

single cell isolation, human lung organoids, single cell RNA sequencing, methanol fixation, rehydration of methanol fixed cells

CREATED

Jul 26, 2022

LAST MODIFIED

Sep 27, 2022

PROTOCOL INTEGER ID

67633

PARENT PROTOCOLS

Part of collection

Collection: State-of-the-Art Analytical Methods of Viral Infections in Human Lung Organoids

GUIDELINES

This protocol describes the processing of human alveolar-like organoids which have been grown according to Youk et al., 2020. https://doi.org/10.1016/j.stem.2020.10.004

MATERIALS TEXT

A	B	C	D
**Substance**	**Company**	**Order number**	**Concentration**
GlutaMax 100x	invitrogen	35050-038	5 mL/500 mL
Hepes	invitrogen	15630-056	5 mL/500 mL
Advanced DMEM/F12	invitrogen	12634-034	1x

Composition of base medium

A	B	C
**Substance**	**Company**	**Order number**
TrypLE™ Express Enzyme (1x)	Gibco	12604013
"Sterican® G 27 x 3/4" " / ø 0,40 x 20 mm, grey" needle	Braun	4657705
Omnifix®-F syringe	Braun	9161406V
5 mL Polystyrene Round-Bottom Tube with Cell-Strainer Cap (FACS tubes)	Falcon	352235
Flowmi® cell strainer	VWR	734-5950

Materials for single cell isolation of lung organoids

A	B	C
**Substance**	**Company**	**Order number**
Dulbecco's Phosphate-Buffered Saline (DPBS)	Thermo Fisher Scientific	14190144
UltraPure Bovine Serum Albumin (BSA, 50 mg/mL	Thermo Fisher Scientific	AM2616
Methanol (for HPLC, ≥99.9%)	Millipore/Sigma	34860-100ml
Protector RNAse Inhibitor (40 U/µL)	Millipore/Sigma	3335399001
SSC Buffer 20X Concentrate	Millipore/Sigma	S6639-1l
DL-Dithiothreitol Solution BioUltra, for molecular biology	Millipore/Sigma	43816

Materials for optional methanol fixation

Wash-Resuspension Buffer: 0.04% BSA + 1 mM DTT + 0.2 U/µL RNase Inhibitor in 3X SSC Buffer (diluted in RNAse free water)

DISCLAIMER:

Informed written consent was obtained from all volunteers and the study was approved by the Charité Ethics Committee (project 451, EA2/079/13).

Single cell dissociation

1 Remove organoid medium from two wells and add **1 mL** cold base medium per well (300,000 cells per well is a good amount to have sufficient cells even after the dissociation process.

2 Transfer organoids with a 1000 µL pipette to a 2 mL reagent tube and spin down at **300 x g, 4°C, 00:05:00** . 5m

Very carefully discard the supernatant with a 1000 µL pipette.

3

4 Resuspend the cell pellet in 1 mL TrypLE Express/well (initally combined).

15m

5 Incubate at 37 °C for 00:15:00 carefully vortex every 5 mins, stop the reaction with 1 mL of base medium.

5m

6 Spin down at 300 x g, 4°C, 00:05:00 .

7 Very carefully discard the supernatant.

8 Resuspend the cell pellet in 1 mL cold base medium.

9 Resuspend the whole supernatant 3 times with 27G needle and 1 mL syringe.

10 Filter cells through the cap of FACS tubes (40 µm pore size).

11 Count cells with the use of Trypan Blue staining. Make sure a single cell solution is provided. According to 10x Genomics recommendation a high-quality cell suspension has high cell viability (>90% ideally, >70% is acceptable), low cell debris and low cell clumping.

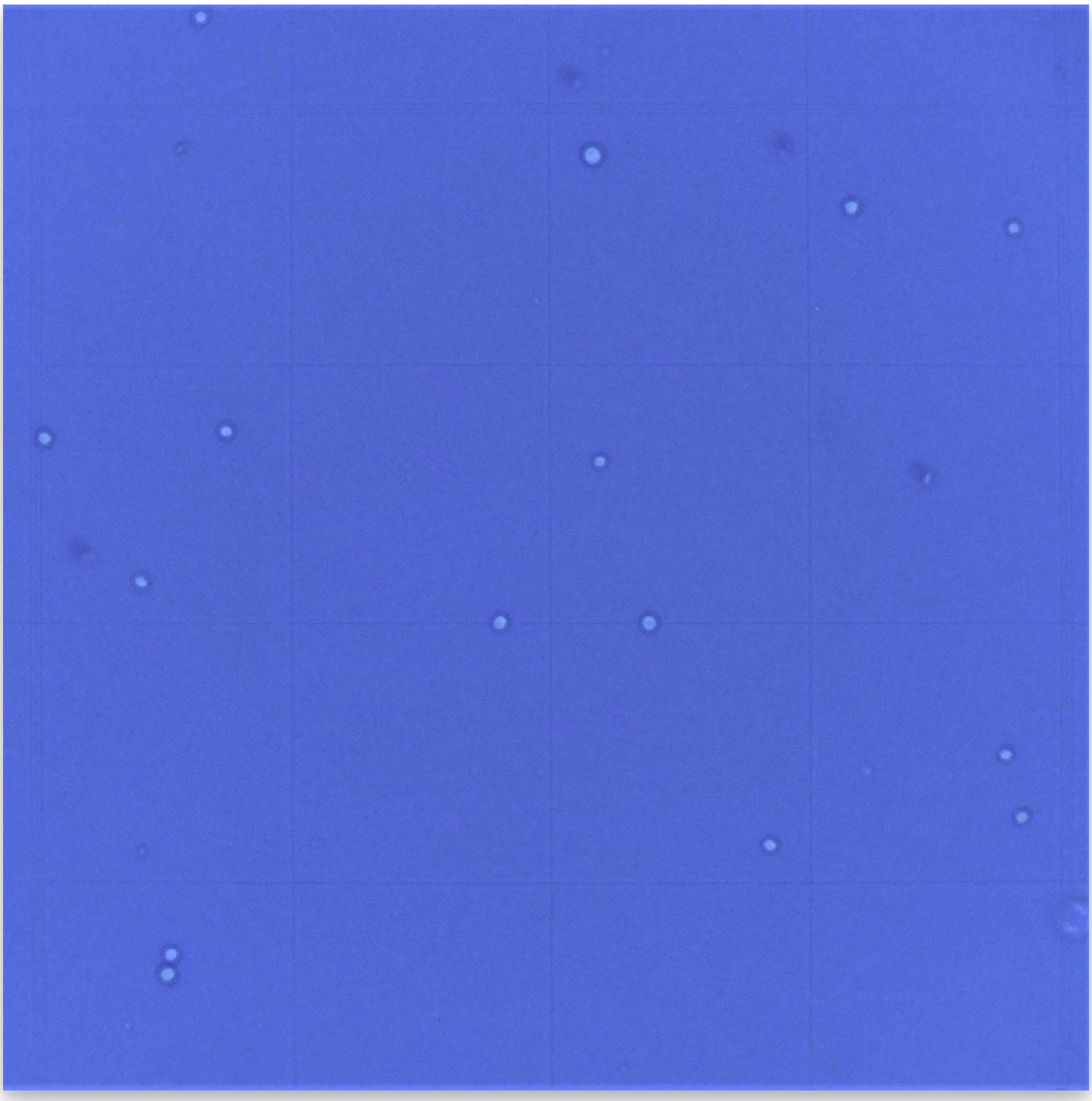

Exemplary Single Cell solution without cell clumps and with good cell viability

12 Proceed with methanol fixation **or** prepare samples immediately according to 10x Genomics protocol for RNA-Sequencing **or** flow cytometry.

For single cell RNA-sequencing samples can be exported to S2 level after GEM-RT incubation (see 10x Genomics protocol: Chromium Next GEM Single Cell 3' Reagent Kits v3.1User Guide).

10x Genomics kits used: Chromium Next GEM Single Cell 3' GEM, Library & Gel Bead Kit v3.1, 16 rxns (1000121), Chromium Next GEM Chip G Single Cell Kit (1000120) and Single Index Kit T Set A (1000213).

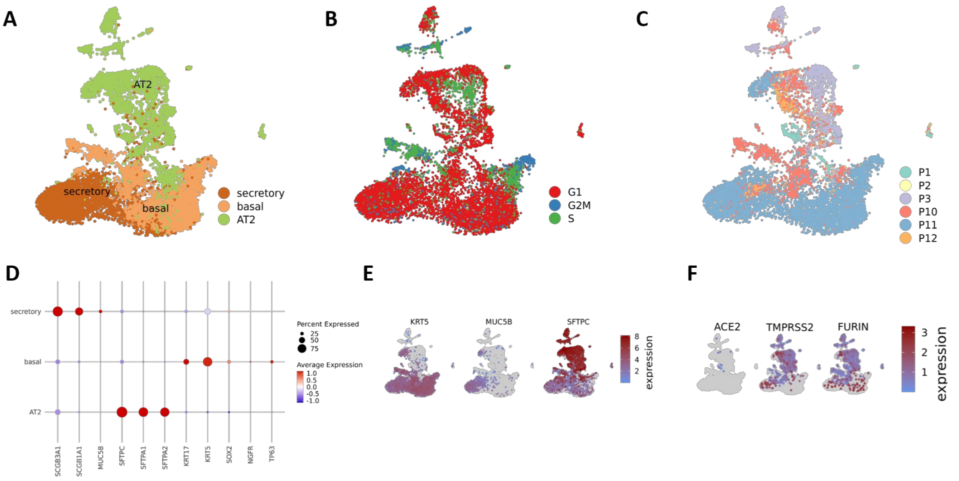

UMAP embedding of human alveolar-like organoids (n = 6; P1-P3 and P10-P12) shows (A) the cell types present in the organoids, which are AT2, basal, and secretory cells. (B) Cell cycle phase of each cell in either G2M, S or G1 phase and (C) individual donor composition is displayed. (D and E) The marker genes needed for cell type identification are shown at single cell resolution as well as (F) the expression of the SARS-CoV-2 host factors *ACE2*, *TMPRSS2*, and *FURIN*.

The Cell Ranger Software Suite (Version 3.1.0) was used to process raw sequencing data with the GRCh38 reference for the control samples, and GRCh38 augmented by the SARS-CoV-2 (NC_045512.2). Viral transcripts including 3'UTR sequences were extracted from the Genbank records and added to the Gencode v33 reference. We used CellBender to remove background RNA and scrublet to identify doublets. Single-cell RNA sequencing data analysis was performed in R (version 3.6.3) with Seurat (version 3.2.1). Cells with at least 500 and less than 5000 detected genes and less than 10% mitochondrial content were combined from each library and library depth (total number of UMIs) was regressed out when scaling data. We additionally regressed out cell cycle scores calculated with Seurat's CellCycleScoring function. After automated clustering, DoubletFinder was used to again identify likely cell doublets, and cluster annotation was performed with Seurat's 'TransferData' workflow using the Human Lung Cell Atlas reference dataset, using only epithelial cells for the organoid data.

Methanol fixation (optional) 40m

13

In case of scRNA-sequencing: Ideally methanol fixation should be avoided and the samples should be immediately processed for library preparation according to the 10x Genomics protocol (Chromium Next GEM Single Cell 3' Reagent Kits v3.1 (Dual Index) with Feature Barcode technology for Cell Multiplexing User Guide).

14 Centrifuge the dissociated cells at **300 x g, 4°C, 00:05:00** . 5m

15 Remove the supernatant without disrupting the cell pellet.

16 Add **1 mL** 1x PBS and gently resuspend pellet 10 times with 1000 µL pipette tip or until cells

are resuspended. Each tube should have 1-2 x10^6 cells (1 well of organoids usually contains up to 0.5 x10^6 cells).

17 Repeat washing steps 14, 15 and 16.

18 5m

Centrifuge at **300 x g, 4°C, 00:05:00** .

19 Remove the supernatant without disrupting the cell pellet.

20 Add **200 µL** chilled 1x PBS/1x10^6 cells and gently resuspend pellet 10x with 1000 µL pipette tip or until cells are resuspended.

21 Add **800 µL** chilled 100% methanol/1x10^6 cells. To avoid clumping of cells, add methanol drop by drop while gently stirring the cell suspension with the pipette tip in the tubes.

> Scale up the volumes of 1x PBS and methanol if using >1 x 10^6 cells.

22 Incubate for **00:30:00** at **-20 °C** . 30m

23 Export from the BSL3 and store fixed cells either at **-20 °C** or **-80 °C** (stability has been shown for both termperatures for up to 6 weeks).

> Before proceeding with the 10x Genomics protocol for RNA Sequencing the cells need to be rehydrated.

Rehydration of methanol-fixed cells 10m

24 Place the microcentrifuge tube containing the methanol-fixed cells on ice to equilibrate to **4 °C** for **00:05:00** . 5m

25 Centrifuge fixed cells at **1000 x g, 4°C, 00:05:00** . 5m

26 Remove the supernatant without disrupting the cell pellet.

27 Based on starting cell concentration and assuming ~50% cell loss, add an appropriate volume of Wash-Resuspension Buffer to obtain a concentration of 700-1,200 cells/µL. Gently pipette mix using a regular-bore pipette tip until a single cell suspension is achieved.

28 Pass the sample through a 40 µm Flowmi Cell.

29 Count cells.

30 Proceed immediately with the 10x Genomics Single Cell protocols. Delay in proceeding may result in RNA loss.
