# Supplementary material for: State-of-the-art analytical methods of viral infections in human lung organoids
Source: PLoS One. 2022 Dec 20;17(12):e0276115. doi: 10.1371/journal.pone.0276115 (PMC9767351; doi:10.1371/journal.pone.0276115)
Supplement: S6 File — (PDF) [file pone.0276115.s006.pdf]

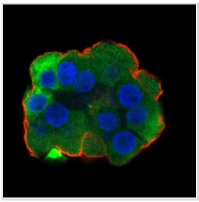

ceattaen

Fixation, Immunohistochemistry and in situ Hybridization of Human Lung Organoids V.(ceattaen)

In 1 collection

Morris Baumgardt[1], Maren Hülsemann,[2], Katharina Hellwig[1], Alina Langenhagen[3], Anne Voss[3], Simon Dökel[3], Achim D. Gruber[3], Stefan Hippenstiel[1], Andreas C. Hocke[1], Katja Hönzke[1]

[1]Charité – Universitätsmedizin Berlin, corporate member of Freie Universität Berlin and Humboldt Universität zu Berlin, Department of Infectious Diseases and Respiratory Medicine, Charitéplatz 1, 10117 Berlin, Germany.;

[2]Berlin Institute of Health at Charité (BIH), BIH QUEST Center for Responsible Research, Berlin, Germany;

[3]Department of Veterinary Pathology, Freie Universität Berlin, Berlin, Germany

1 Works for me

Reserved DOI:
10.17504/protocols.io.e6nvwk5p2vmk/v3

morris.baumgardt

DISCLAIMER

Informed written consent was obtained from all volunteers and the study was approved by the Charité Ethics Committee (project 451, EA2/079/13).

ABSTRACT

This protocol describes the fixation of infected human alveolar-like organoids, as well as the sample preparation for immunohistochemistry and immunohistology of infected tissue from alveolar-like organoids.

PROTOCOL INFO

Morris Baumgardt, Maren Hülsemann,, Katharina Hellwig, Alina Langenhagen, Anne Voss, Simon Dökel, Achim D. Gruber, Stefan Hippenstiel, Andreas C. Hocke, Katja Hönzke . Fixation, Immunohistochemistry and in situ Hybridization of Human Lung Organoids . **protocols.io**
https://protocols.io/view/fixation-immunohistochemistry-and-in-situ-hybridiz-ceattaen
Version created by morris.baumgardt

COLLECTIONS

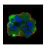

Collection: State-of-the-Art Analytical Methods of Viral Infections in Human Lung Organoids

KEYWORDS

immunohistochemistry, human lung organoids, fixation of organoids, in situ hybridization

CREATED

Jul 26, 2022

LAST MODIFIED

Sep 27, 2022

PROTOCOL INTEGER ID

67635

PARENT PROTOCOLS

Part of collection

Collection: State-of-the-Art Analytical Methods of Viral Infections in Human Lung Organoids

GUIDELINES

This protocol describes the processing of human alveolar-like organoids which have been grown according to Youk et al., 2020. https://doi.org/10.1016/j.stem.2020.10.004.

MATERIALS TEXT

A	B	C	D
**Substance**	**Company**	**Order number**	**Concentration**
GlutaMax 100x	invitrogen	35050-038	5 mL/500 mL
Hepes	invitrogen	15630-056	5 mL/500 mL
Advanced DMEM/F12	invitrogen	12634-034	1x

Composition of base medium

A	B	C
**Substance**	**Company**	**Order number**
Formaldehyde solution 4%, buffered, pH 6.9	Sigma Aldrich	1004965000
Roticlear	Carl Roth	A538.5

Materials for organoid fixation

Bufers, assays and special equipment used:

10 mM TRIS + 1 mM EDTA buffer pH 9.0

1.2 g TRIS (1 M = 121.14 g/L)
0.29 g EDTA (1 M = 292.2 g/L)
–> Ø 1 L Aqua bidest (store at RT)

Dilution medium for IF

50 mL PBS 0.01 M
0.5 g BSA
2 drops Tween 20
–> Store at 4°C

0.01 M PBS

0.9 g NaH_2PO_4
7.75 g Na_2HPO_4
43.6 g NaCl
–> 5 L Aqua bidest (store at RT)

A	B	C
**Description**	**Company**	**Order number**
Tissue-Tek VIP	Sakura	
Modular Tissue Embedding Center EC 350-2	Myr	
Epredia HM 325 Rotary Microtome	fisher scientific	15340735
COP 30 Cooling plate	VWR	632-7014
Robotic coverslipper CV5030	Leica	14 0478 80100
StarFrost® Adhesive Slides	VWR	KNITVS11371077FKA
Ethanol denatured with 2 % isopropyl	Berkel	
Xylene technical grade	Th. Geyer	399-5L-PE
Hemalum solution acid acc. to Mayer	Roth	T865.1
Waldeck, Eosin 2 %	Biosystems	84-0023-00

Devices/materials for embedding, sectioning and HE staining

A	B	C	D
**Substance**	**Company**	**Order number**	**RRID**
Formaldehyde solution 4%, buffered, pH 6.9	Sigma Aldrich	1004965000	
goat-anti-ACE2, 1:100 IF	R&D	AF933	AB_355722
donkey anti-Goat IgG (H+L) Cross-Adsorbed Secondary Antibody, Alexa Fluor 488 1:2000	Thermo Fisher	A-11055	AB_2534102
FluoTag®-Q anti-SARS-CoV-2 Nucleocapsid, Sulfo-Cyanine 3 1:100	NanoTag Biotechnologies	N3601-SC3-L	
DAPI 4',6-Diamidine-2'-phenylindole dihydrochloride 1:100	Roche Diagnostics	10236276001	

Materials and antibodies for immunohistology used for host factor and virus detection

A	B	C
**Substance**	**Company**	**Order number**
Xylen	Chemsolute	371.500
Ethanol	Roth	9065.4
Fluoromount with DAPI	Roth	HP20.1
ViewRNA ISH Tissue Kit (contains protease, probe diluent, amplifier mix, fast red, washing buffer etc)	Invitrogen (Thermo Fisher Sci)	19931
CoV2 Probe	Invitrogen (Thermo Fisher Sci)	VPNKRHM
EF1a Probe	Invitrogen (Thermo Fisher Sci)	VA1-10418-VT
dapB Probe	Invitrogen (Thermo Fisher Sci)	VF1-11712-VT

Materials and antibodies for immunohistochemistry (RNAview)

A	B	C
**Substance**	**Company**	**Order number**
ACE2 Probe	ACD biotechne	848151-C2
RNAscope® 3-plex Positive Control Probe-Hs	ACD biotechne	320861
RNAscope® 3-plex Negative Control Probe	ACD biotechne	320871

Antibodies for immunohistochemistry (RNAscope)

A	B	C
**Device**	**Company**	**Order number**
Fisherbrand™ Isotemp™ General Purpose Heating and Drying Ovens	fisher scientific	15-103-0503
Staining cuvette	Roth	2287.1
Magnetic Stirrer with heater	neolab	D-6010
Waterproof thermometer	VWR	TD 131
Ultra-accurate probes	VWR	PTZ 341

Additional material used for immunohistochemistry (RNAscope)

DISCLAIMER:

Informed written consent was obtained from all volunteers and the study was approved by the Charité Ethics Committee (project 451, EA2/079/13).

Fixation 25m

1 Remove the organoid medium from the organoid containing well. Organoids should be collected from minimum two wells of a 24-well plate, to obtain a sufficient amount of organoids for staining.

2 Add **1 mL** cold base medium and collect Cultrex with organoids in a tube, flush well with **1 mL** base medium.

3 Place tubes with organoids at 4 °C for 00:05:00 (to dissolve Cultrex). 5m

4 Centrifuge for 300 x g, 4°C, 00:05:00 . 5m

5 Remove supernatant, add 4% Formaldehyde solution (FA) and pipette up and down.

6

Store over night at 4 °C in the BSL3 lab.

7 Export vessel from the BSL3 with organoid and FA, centrifuge for 300 x g, 4°C, 00:05:00 remove FA and add fresh 4% FA. 5m

8 Store over night at 4 °C .

9 Centrifuge for 300 x g, 00:05:00 and remove FA. 5m

10 Resolve pellet in PBS and store at 4 °C or proceed with embedding.

11 Centrifuge for 300 x g, 00:05:00 and remove PBS. 5m

12 Resolve pellet in 100 µL tissue Tek embedding medium (Histogel), pipette a drop on parafilm, place on ice and upon solidification place in the embedding cassette (act fast).

13 Close cassette, store in PBS, proceed with paraffin embedding and sectioning (sections: 2 µm).

Paraffin embedding and sectioning 25m

14 Put cassette into the automatic embedding machine and start the following program:

A	B	C	D	E	F	G
**Step**	**Solution**	**Concentration (%)**	**Duration (min:sec)**	**Temperature (°C)**	**D/V**	**Mix**
**1**	Formalin	4	0:00	–	ON	OFF
**2**	Water		0:30	40	ON	OFF
**3**	Alcohol	70	1:00	40	ON	Slow
**4**	Alcohol	80	1:00	40	ON	Slow
**5**	Alcohol	96	1:00	40	ON	Slow
**6**	Alcohol	96	1:00	40	ON	Slow
**7**	Alcohol	100	1:00	40	ON	Slow
**8**	Alcohol	100	1:00	40	ON	Slow
**9**	Xylene		1:00	40	ON	Slow
**10**	Xylene		1:00	40	ON	Slow
**11**	Paraffin		1:00	60	ON	OFF
**12**	Paraffin		1:00	60	ON	OFF
**13**	Paraffin		1:00	60	ON	OFF
**14**	Paraffin		1:00	60	ON	OFF

Tissue-Tek VIP embedding program

15 In the following, after embedding, the samples are sectioned and mounted on adhesive slides. Sections of 2 µm each are prepared. Sections 1,5,9,13 are stained HE (following table) to see in which stages organoids are present, the rest is kept as blank section.

A	B	C
**Step**	**Solution**	**Duration (min:sec)**
**1**	Xylene	2x 2:00
**2**	Xylene	1x 3:00
**3**	96% ethanol	0:30
**4**	80% ethanol	0:30
**5**	70% ethanol	0:30
**6**	Water	1:00
**7**	Hematoxylin	8:00
**8**	Water	0:05
**9**	70% ethanol	0:10
**10**	80% ethanol	0:30
**11**	96% ethanol	0:45
**12**	100% ethanol	1:00
**13**	100% ethanol	1:00
**14**	Xylene	4x 1:00

HE staining steps

Immunohistology staining 3h 26m

16

Dewaxing over night at **60 °C** in Roticlear.

17 Put the slide with organoids three times for **00:15:00** in Roticlear at **60 °C** (use fresh Roticlear for every step). 15m

18 **00:15:00** 100% Ethanol. 15m

19 **00:01:00** 100% Ethanol. 1m

20 **00:10:00** 96% Ethanol. 10m

21 **00:05:00** 80% Ethanol. 5m

22 00:05:00 70% Ethanol. 5m

23 00:05:00 50% Ethanol. 5m

24 Shake 3x 00:05:00 in [M]0.01 Molarity (M) PBS (100 U/min). 5m

25 Antigen-Retrival for 00:30:00 in a steam bath: Tris-EDTA buffer. 30m

26 Let cool down for approx. 00:30:00 . 30m

27 Shake 3x 00:05:00 in [M]0.01 Molarity (M) PBS (100 U/min). 5m

28 00:15:00 permeabilisation in 1% Triton (in [M]0.01 Molarity (M) PBS) (on shaker 100 U/min). 15m

29 Shake 3x 00:05:00 in [M]0.01 Molarity (M) PBS (100 U/min). 5m

30 Blocking for 00:30:00 in 5% appropriate serum (in dilution medium), ca. 70 µL per tissue slice. 30m

31 Flush carefully with [M]0.01 Molarity (M) PBS (Pasteur pipette).

32 Dilute primary antibody in dilution medium and incubate over night at 4 °C (wet chamber).

33 Flush carefully with [M]0.01 Molarity (M) PBS (Pasteur pipette).

34 Shake 3x 00:05:00 in [M]0.01 Molarity (M) PBS (100 U/min). 5m

35 Dilute secondary antibody in dilution medium and incubate over night at 4 °C (wet chamber).

36 Flush carefully with [M]0.01 Molarity (M) PBS (Pasteur pipette).

37 Shake 3x 00:05:00 in [M]0.01 Molarity (M) PBS (100 U/min). 5m

38 Incubate 00:05:00 with DAPI. 5m

39 Shake 3x 00:05:00 in [M]0.01 Molarity (M) PBS (100 U/min). 5m

40 Embedding with Mowiol® and coverslip, let air dry for 00:10:00 and seal edges with nail polish. 10m

41 Immunofluorescence is analyzed by spectral confocal microscopy using a LSM 780 [Carl-Zeiss, Jena, Germany]. Based on a spectral image lambda stack, linear unmixing of tissue autofluorescence and overlapping spectra of fluorochromes is performed using ZEN 2012 software (Carl-Zeiss, Jena, Germany). To reveal lung and cell morphology, images are combined with Differential Interference Contrast (DIC). All image sets are acquired using optimal configuration regarding resolution and signal to noise ratio. Images are processed using ZEN 2012.

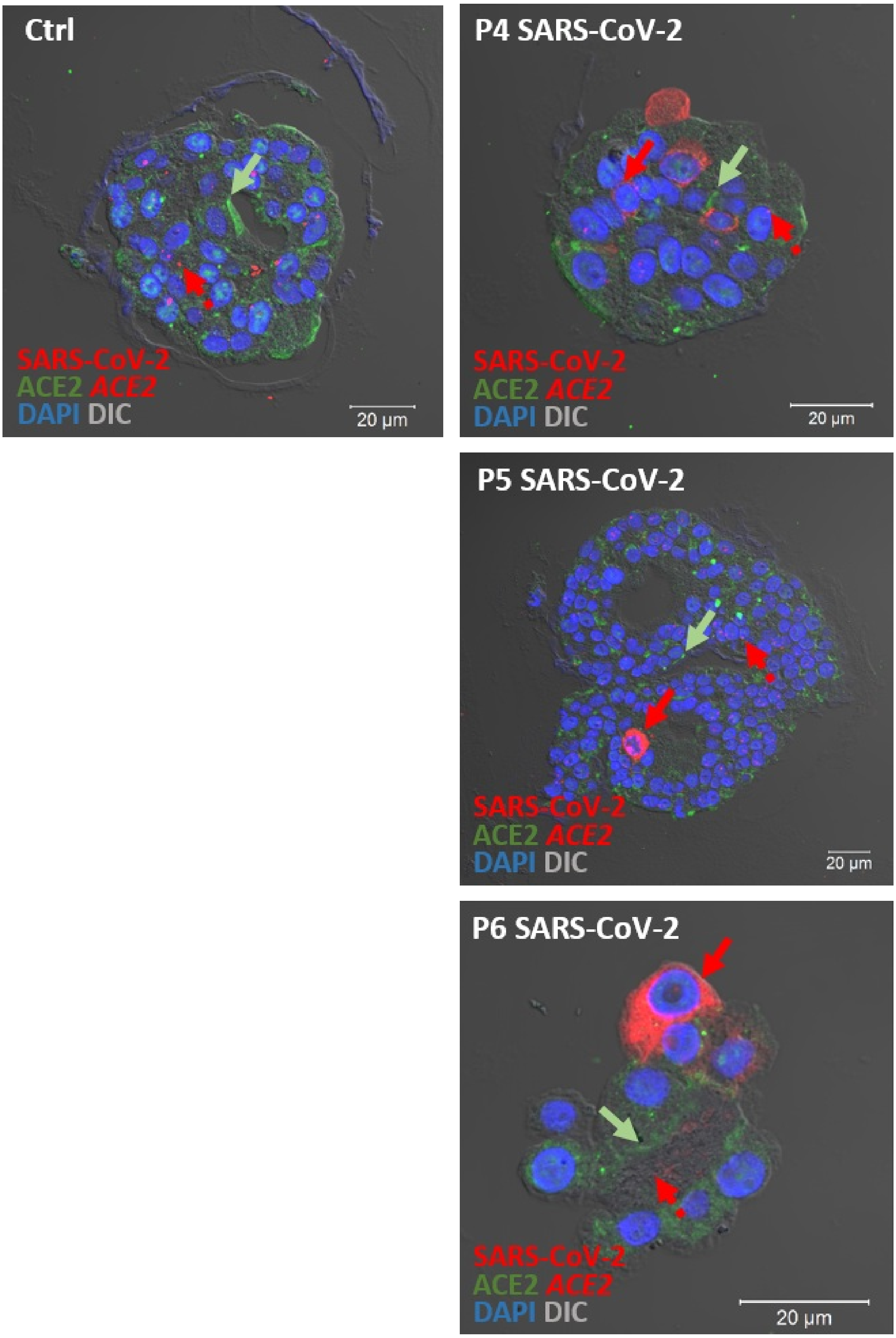

Representative immunostainings for exemplary mock- (left panel) and SARS-CoV-2-infected (right column) human alveolar-like organoids. Shown are immunostainings for SARS-CoV-2 (N-protein, red), ACE2 (green) and *ACE2* mRNA expression (red dots, see *in situ* hybridization - step 55) 24 h post infection (MOI = 1). Arrows indicate either cells positive for SARS-CoV-2 (red arrows) or areas of particularly high ACE2 expression (protein: green arrows, mRNA: red dotted arrows). Cell nuclei are visualized by DAPI stain (blue). Scale bars = 20 µm.

Viral in situ hybridization 1d 5h 35m 45s

42 We used a maximum of 12 slides at a time for the procedure. The size of the FFPE section should not exceed 2/3 of the slide and at least 5 mm away from each slide boarder.

43 Baking:

43.1 Bake the slides for **01:00:00** at **60 °C +-1°C** in a dry oven. 1h

43.2 Direct after baking remove paraffin with a tissue as much as you can, but do not touch the tissue.

44 Deparaffinization:

44.1 Pour **200 mL** of xylene into a staining dish.

44.2 Transfer the baked slides to the staining dish and incubate the slides at room temperature for **00:05:00** . Agitate frequently by moving the rack up and down. 5m

44.3 Repeat the xylene washing with fresh xylene for 2 times.

44.4 Remove the slides from the xylene and wash the slides twice with **200 mL** of 100% ethanol for **00:05:00** with frequent agitation. 5m

44.5 Remove the slides from the ethanol and place them face up on a paper towel to air dry for **00:05:00** at room temperature. 5m

45 Hydrophobic barrier:

45.1 Before using the hydrophobic barrier pen dap it on a paper towel several times to ensure proper flow of the hydrophobic solution.

45.2 To create a hydrophobic barrier draw a rectangle around the organoid section, repeat it 2-4 times to ensure a solid seal.

45.3 Let the barrier dry for ~ **00:25:00** (until it is really dry). 25m

46 Heat Pretreatment:

46.1 Heat the 1x Pretreatment Solution to **90 °C to 95°C** in a porcelain staining jar (do not boil the Solution).

46.2 Incubate the slides for **00:10:00** at **90 °C to 95°C** in the 1x Pretreatment Solution (time depends on tissue sample). 10m

46.3 Remove the slide from the jar and submerge it directly into a clear staining dish containing **200 mL** of full desalted autoclaved water (VE water), at this point the slides should not dry out.

46.4 Wash for **00:01:00** with frequent agitation. 1m

46.5 Repeat the wash step 46.3 and 46.4 one more time with **200 mL** of fresh VE water.

46.6 Transfer the slides to a clear staining dish containing [M]**0.01 Molarity (M)** PBS.

47 Protease digestion and fixation:

47.1 Dilute the Protease (all in ViewRNA Tissue Kit) 1:100 in pre warmed 1X PBS (40 °C) and briefly vortex to mix.

47.2 Remove each slide out of the dish and flick it to remove excess PBS. Do not let the slides dry out.

47.3 Place each slide face up on an object carrier box (OCB; pre tempered to 40 °C / pre wetted with VE water) and immediately add 200 µL of the working protease solution onto the tissue section. Make sure that the tissue section is covered with working protease solution.

47.4 Incubate the OCB in an isotemp oven (Thermo Fisher) at 40 °C for 00:20:00 (Time depends on organoid sample). 20m

47.5 After incubation decant the working protease solution from the slides and wash the slides two times with PBS in a staining dish for 00:01:00 . 1m

47.6 Remove each slide out of the dish and flick it to remove excess PBS. Do not let the slides dry out.

47.7 Place each slide on a paper towel under a fume hood and incubate with 4% PFA for 00:05:00 at room temperature. 5m

47.8 After incubation decant the PFA from the slides and wash the slides two times with PBS in a staining dish for 00:01:00 . 1m

48 Hybridization:

48.1 Dilute the viewRNA probe set 1:40 in pre warmed probe set diluent and briefly vortex to mix. For positive control we use a *EF1α* probe set (also possible is *ACTB* or *GAPDH*) and for negative control we use a *DapB* probe set.

48.2 Remove each slide out of the dish and flick it to remove excess PBS. Do not let the slides dry out.

48.3 Place each slide face up on an OCB and immediately add **200 µL** of the diluted probe set solution onto the tissue section. Make sure that the tissue section is covered with solution.

48.4 Incubate the OCB in an isotemp oven at **40 °C** for **02:00:00** . 2h

48.5 Prepare **2 L** wash buffer with VE water, **18 mL** wash comp1 and **5 mL** wash comp2.

48.6 After incubation decant the probe set solution and wash the slides in a staining dish with wash buffer for **00:02:00** . Repeat washing for 2 times more with fresh washing buffer. 2m

48.7 Optionally the slides can be stored at this point in **200 mL** storage buffer (**60 mL** comp2 and **140 mL** VE water) for **24:00:00** at RT. 1d

48.8 After storage the slides must be washed again 3 times with wash buffer.

49 Pre amplifier hybridization:

49.1 Pre warm the pre amplifier mix and swirl it to mix the solution.

49.2 Remove each slide out of the dish and flick it to remove excess liquid. Do not let the slides dry out.

49.3 Place each slide face up on an OCB and immediately add **200 µL** of the pre amplifier mix onto the tissue section. Make sure that the tissue section is covered with solution.

49.4 Incubate the OCB in an isotemp oven at **40 °C** for **00:25:00** . 25m

49.5 After incubation decant the pre amplifier mix and wash the slides in a staining dish with wash buffer for 00:02:00 . Repeat washing for 2 times more with fresh washing buffer. 2m

50 Amplifier hybridization:

50.1 Pre warm the amplifier mix and swirl it to mix the solution.

50.2 Remove each slide out of the dish and flick it to remove excess liquid. Do not let the slides dry out.

50.3 Place each slide face up on an OCB and immediately add 200 µL of the amplifier mix onto the tissue section. Make sure that the tissue section is covered with solution.

50.4 Incubate the OCB in an isotemp oven at 40 °C for 00:15:00 15m

50.5 After incubation decant the amplifier mix and wash the slides in a staining dish with wash buffer for 00:02:00 . Repeat washing for 2 times more with fresh washing buffer. 2m

51 Label Probe 1-AP hybridization:

51.1 After incubation decant the amplifier mix and wash the slides in a staining dish with wash buffer for 00:02:00 . 2m

51.2 Repeat washing for 2 times more with fresh washing buffer.

51.3 Remove each slide out of the dish and flick it to remove excess liquid. Do not let the slides dry out.

51.4 Place each slide face up on an OCB and immediately add **200 µL** of the label probe 1-AP solution onto the tissue section. Make sure that the tissue section is covered with solution.

15m

51.5 Incubate the OCB in an isotemp oven at **40 °C** for **00:15:00** .

3m

51.6 After incubation decant the amplifier mix and wash the slides in a staining dish with wash buffer for **00:03:00** .

51.7 Repeat washing for 2 times more with fresh washing buffer.

52 Fast Red Substrate:

5m

52.1 Remove the slides from the dish and place them face up on a paper towel, immediately add **200 µL** of the AP Enhancer Solution to each slide and incubate for **00:05:00** .

52.2 Decant the AP Enhancer Solution after incubation, place each slide face up on an OCB and add directly **200 µL** of fresh prepared fast red mix (for **2.5 mL** fast red buffer add **40 µL** fast red substrate1, vortex then add **40 µL** substrate2, vortex at least add **40 µL** substrate3, vortex).

1h

52.3 Incubate the OCB at RT in the dark for **01:00:00** .

1m

52.4 After incubation decant the fast red mix and wash the slides in a staining dish with PBS for **00:01:00** .

52.5 Repeat washing for one time more with fresh PBS.

53 Counter staining:

53.1 Optionally counter stain for **00:00:45** with Gill's hematoxylin. 45s

53.2 Wash with water for **00:05:00** . 5m

53.3 Mount the slides with fluoromount (Roth) by using a robotic coverslipper.

54

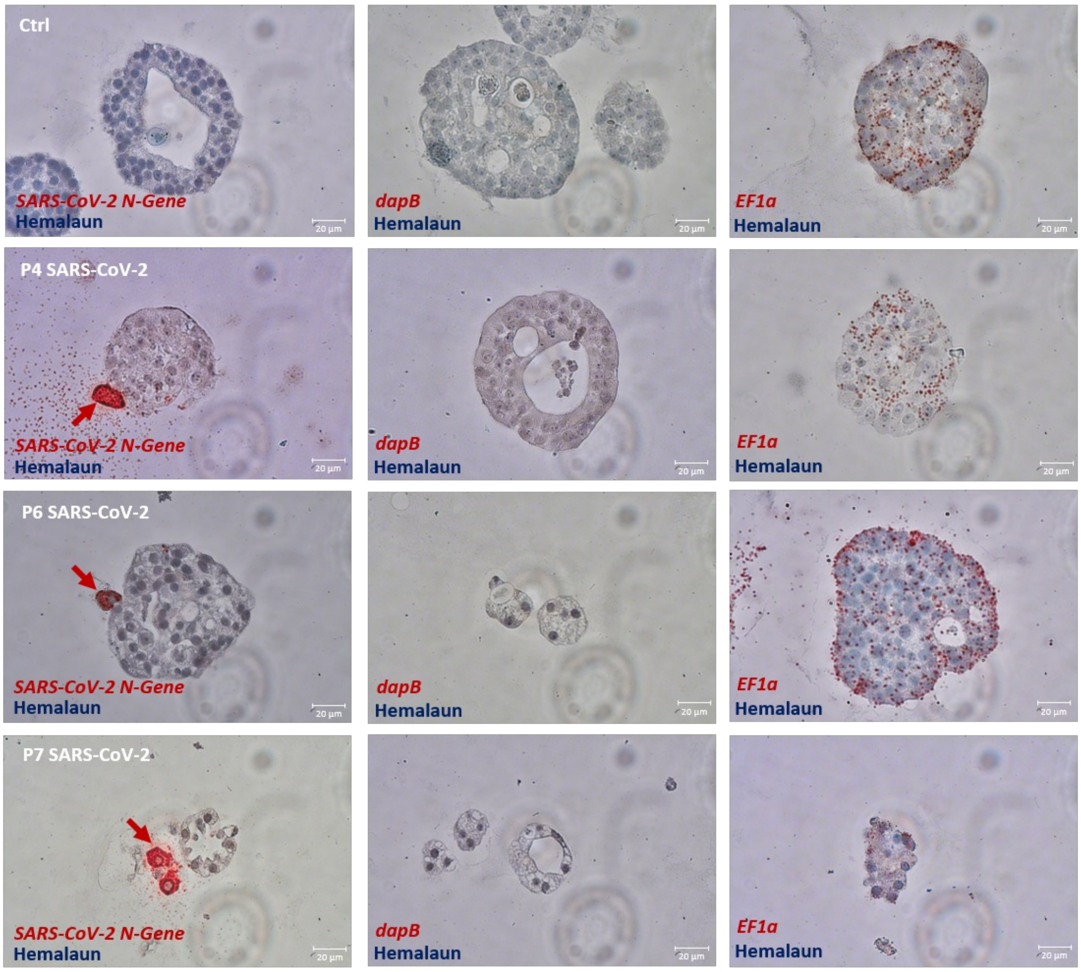

In situ **hybridization of human alveolar-like organoids (three donors) shows SARS-CoV-2 mRNA expression (left column, red).** ***DapB*** **and** ***EF1a*** **served as negative respectively positive control. Red arrows indicate cells positive for SARS-CoV-2. Scale bars = 20 µm.**

ACE2 in situ hybridization 1d 5h 35m 45s

55 For *ACE2 in situ* hybridization proceed with the "RNAscope® Multiplex Fluorescent Reagent Kit v2 Assay":

USM-323100_Multiplex_Fluorescent_v2_User_Manual_10282019.pdf

The following changes were made using the USM-323100 protocol:

Page 14, step 5: a third xylene incubation step is implemented (5 min at RT).
Page 15, target retrieval: a digital heat plate is used instead of a steamer.
Page 17: a staining cuvette is used instead of the slide holder.

After finishing the steps for "FFPE sample preparation and pretreatment", continue with chapter 4 on page 28.

Page 31: Opal is used with a dilution of 1:1500.

Exemplary results are shown at **step 41**.
