# Supplementary figures and images for: State-of-the-art analytical methods of viral infections in human lung organoids
Source: PLoS One. 2022 Dec 20;17(12):e0276115. doi: 10.1371/journal.pone.0276115 (PMC9767351; doi:10.1371/journal.pone.0276115)

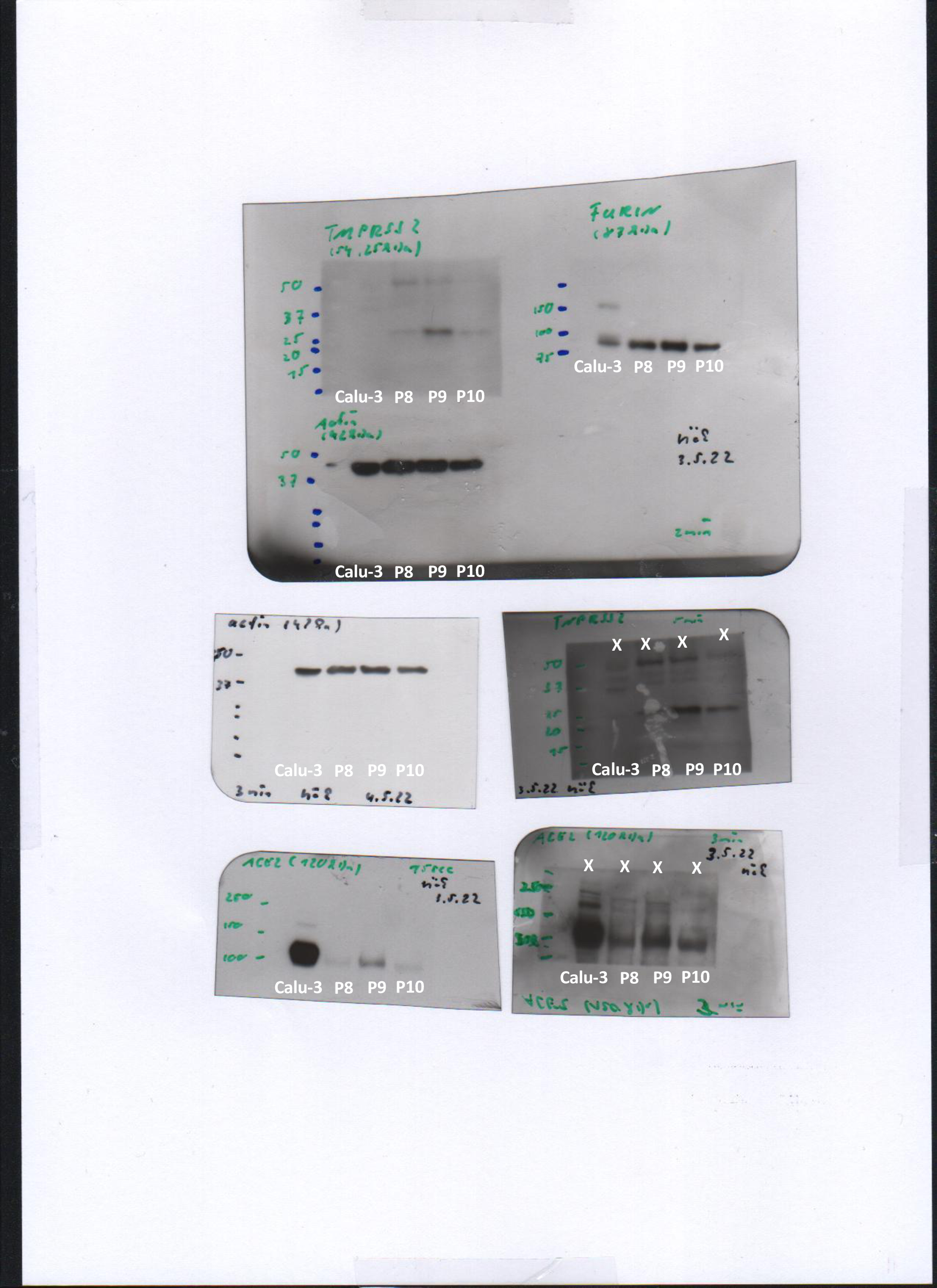

Supplement: S1 Raw images — (TIF) [file pone.0276115.s007.tif]
